# Ethylene induced plant stress tolerance by *Enterobacter* sp. SA187 is mediated by 2‐keto‐4‐methylthiobutyric acid production

**DOI:** 10.1371/journal.pgen.1007273

**Published:** 2018-03-19

**Authors:** Axel de Zélicourt, Lukas Synek, Maged M. Saad, Hanin Alzubaidy, Rewaa Jalal, Yakun Xie, Cristina Andrés-Barrao, Eleonora Rolli, Florence Guerard, Kiruthiga G. Mariappan, Ihsanullah Daur, Jean Colcombet, Moussa Benhamed, Thomas Depaepe, Dominique Van Der Straeten, Heribert Hirt

**Affiliations:** 1 King Abdullah University of Science and Technology, Division of Biological and Environmental Sciences and Engineering, Thuwal, Kingdom of Saudi Arabia; 2 Institut of Plant Sciences Paris-Saclay (IPS2), UMR 9213/UMR1403, CNRS, INRA, Université Paris-Sud, Université d’Evry, Université Paris-Diderot, Sorbonne Paris-Cité, Orsay, France; 3 King Abdulaziz University, Faculty of Meteorology, Environment and Arid Land Agriculture, Jeddah, Saudi Arabia; 4 Ghent University, Department of Physiology, Laboratory of Functional Plant Biology, Ghent, Belgium; Utrecht University, NETHERLANDS

## Abstract

Several plant species require microbial associations for survival under different biotic and abiotic stresses. In this study, we show that *Enterobacter* sp. SA187, a desert plant endophytic bacterium, enhances yield of the crop plant alfalfa under field conditions as well as growth of the model plant *Arabidopsis thaliana in vitro*, revealing a high potential of SA187 as a biological solution for improving crop production. Studying the SA187 interaction with Arabidopsis, we uncovered a number of mechanisms related to the beneficial association of SA187 with plants. SA187 colonizes both the surface and inner tissues of Arabidopsis roots and shoots. SA187 induces salt stress tolerance by production of bacterial 2-keto-4-methylthiobutyric acid (KMBA), known to be converted into ethylene. By transcriptomic, genetic and pharmacological analyses, we show that the ethylene signaling pathway, but not plant ethylene production, is required for KMBA-induced plant salt stress tolerance. These results reveal a novel molecular communication process during the beneficial microbe-induced plant stress tolerance.

## Author summary

Plants as sessile organisms are facing multiple stresses during their lifetime. Among them, abiotic stresses, such as salt stress, can cause severe crop yield reduction, leading to food security issues in many regions of the world. In order to respond to growing food demands, especially in the context of the global climate change and increasing world population, it then becomes urgent to develop new strategies to yield crops more tolerant to abiotic stresses. One way to overcome these challenges is to take advantage of plant beneficial microbes, defined as plant growth promoting bacteria (PGPB). In this study, we report the beneficial effect of *Enterobacter* sp. SA187 on plant growth under salt stress conditions. SA187 increased the yield of the forage crop alfalfa when submitted to different saline irrigations in field trials. Moreover, using the model plant *Arabidopsis thaliana*, we demonstrate that SA187 mediates its beneficial activity by producing 2-keto-4-methylthiobutyric acid (KMBA), which modulates the plant ethylene signaling pathway. This study highlights a novel mechanism involved in plant-PGPB interaction, and proves that endophytic bacteria can be efficiently used to enhance yield of current crops under salt stress conditions.

## Introduction

Abiotic stresses like salinity, drought or heat negatively affect plant growth and yield and belong to the most limiting factors of agriculture worldwide [1,2]. For example, salinity, known to affect almost one fourth of arable land globally, is a two-phase stress composed of a rapid osmotic and a slower toxic stress, resulting from Na^+^ ion accumulation and loss of K^+^ in photosynthetic tissues [3]. Salt stress reduces the rate of photosynthesis, leading to a decrease of plant growth and crop yield [4]. However, in the context of global climate change and an increasing world population, abiotic stress tolerant crops and sustainable solutions in agriculture are urgently needed to respond to growing food demands [5].

One way to overcome these challenges is to take advantage of plant-interacting microbes [6–8]. Indeed, plants and their rhizosphere host diverse microbial communities, selected from bulk soil [9–11], and beneficial bacteria, defined as plant growth-promoting bacteria (PGPB), can establish symbiotic associations with plants and promote their growth under optimal growth conditions or in response to biotic and abiotic stresses [12–18]. Direct plant growth-promotion mechanisms include the acquisition of nutrients by nitrogen fixation, phosphate and zinc solubilization, or siderophore production for sequestering iron. The modulation of phytohormone levels, such as auxin, ethylene, cytokinin or gibberellin, also largely contributes to the beneficial properties of PGPB [19–21]. Indirect mechanisms comprise the production of antimicrobial agents against plant pathogenic bacteria or fungi, or inducing systemic resistance against soil-borne pathogens [18,22].

Arid regions cover about one quarter of the Earth’s land surface and encompass many of the challenges for increasing agricultural productivity [23]. In contrast to better known dryland farming, desert agriculture can function only when crop plants are irrigated–usually with underground water with various levels of salinity [24]. Those areas face extreme environmental conditions, characterized by high levels of radiation, low rainfall, extreme temperatures, coarse soil which retains very little moisture, as well as low nutrients and typically high natural salinity, which all strongly limit the yield of crops [25]. Although deserts appear to be hardly inhabitable, a wide diversity of organisms has adapted to these extreme conditions. Plants along with their interacting microbial partners have evolved sophisticated mechanisms such as the production of osmoprotectants, reactive oxygen species scavengers or late embryogenesis abundant proteins to monitor the environment and reprogram their metabolism and development [26,27]. Therefore, this particular environment is an ideal reservoir to isolate and identify beneficial bacteria enhancing plant tolerance towards environmental stresses such as drought, heat or salinity [7].

To identify and characterize stress tolerance-promoting bacteria that can increase plant tolerance to abiotic stresses and therefore could be used for improving desert agriculture, we previously isolated and sequenced a number of rhizosphere and endophytic bacterial strains from nodules of desert pioneer plants [28–30]. Here, we report that *Enterobacter* sp. SA187, an endophytic bacterium isolated from root nodules of the indigenous desert plant *Indigofera argentea* [31], significantly increased yield of the agronomically important crop alfalfa (*Medicago sativa*) in field trials under both normal and salt stress conditions, demonstrating that SA187 has a high potential to improve agriculture under desert conditions. To better understand the molecular mechanisms for conveying enhanced stress tolerance of plants, we studied its interaction with *Arabidopsis thaliana*. SA187 could enhance Arabidopsis tolerance to salt stress, and GFP-labeled SA187 colonized surface and inner tissues of Arabidopsis roots and shoots. Moreover, transcriptome analyses uncovered that SA187-induced plant tolerance to salt stress is due to maintenance of photosynthesis and primary metabolism and a reduction of ABA-mediated stress responses. Using different plant hormone related mutants, ethylene sensing was found to play a primary role in SA187-induced salt stress tolerance. Indeed, Arabidopsis mutants impaired in ethylene perception were compromised in their beneficial response to SA187, while mutants deficient in ethylene synthesis remained unaffected. Gene expression analysis of SA187 indicated an upregulation of the methionine salvage pathway upon plant colonization, increasing the production of 2-keto-4-methylthiobutyric acid (KMBA), which is known to be converted into ethylene *in planta* [32]. KMBA alone could mimic the beneficial effects of SA187 on plant salt stress tolerance and 2,4-dinitrophenylhydrazine (DNPH), which specifically precipitates KMBA [33], could abrogate SA187-induced plant stress tolerance. These results unravel a novel communication process during beneficial plant-microbe interactions under stress conditions.

## Results

### *Enterobacter* sp. SA187 increases alfalfa yield in field trials under field conditions

**Fig 1:**
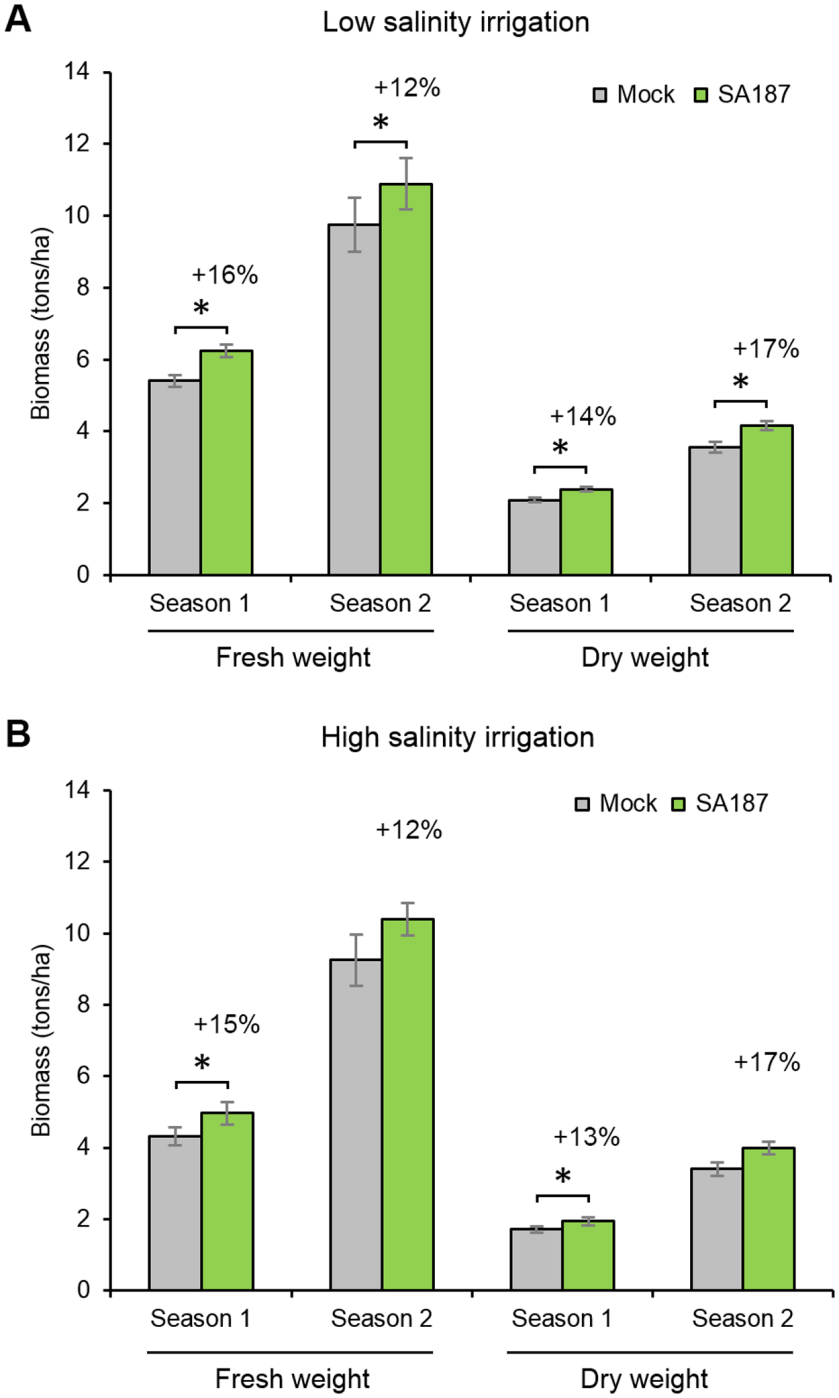
Growth parameters of alfalfa in field trials. (A) Alfalfa fresh and dry weight under low salinity irrigation. (B) Alfalfa fresh and dry weight under high salinity irrigation. Each column represents a mean of harvests from each experimental plot (n = 4 for season 1; n = 3 for season 2). Error bars represent SE. An increase of weight for SA187-treated plants related to Mock is indicated in %. Asterisks indicate a statistical difference based on Factorial ANOVA test followed by least significant difference (LSD) test (* P < 0.05). Meteorological data for field trials are displayed in S1 Fig.

Since SA187 was an outstandingly performing bacterial isolate in a previous screen using Arabidopsis as a model plant [31], we evaluated the potential agronomic use of SA187 as a biological solution for agriculture. Therefore, we tested the beneficial activity of SA187 on different growth parameters of the crop plant alfalfa (*Medicago sativa*), which is largely used as animal feed in different regions of the world. Alfalfa seeds were coated with SA187 and tested in parallel with mock-coated seeds at the experimental field station Hada Al-Sham near Jeddah, Saudi Arabia. A randomized complete block design with a split-split plot arrangement with different replicates was used over two subsequent growing seasons (2015–2016 and 2016–2017). Using low saline water (EC = 3.12 dS·m^-1^) for irrigation, SA187-inoculated alfalfa plants showed an increase of 16 and 12% of fresh weight and 14 and 17% of dry biomass in the two growing seasons, respectively (Fig 1A). Using high saline water (EC = 7.81 dS·m^-1^) for irrigation, a similar beneficial impact on plant growth was observed over the two growing seasons (Fig 1B). However, the growth parameters in the second season were statistically not significant, most likely due to exceptional rainfall in that period (S1 Fig). We concluded that SA187 can efficiently improve crop productivity under extreme agricultural conditions.

### *Enterobacter* sp. SA187 enhances salt tolerance in *Arabidopsis thaliana*

**Fig 2:**
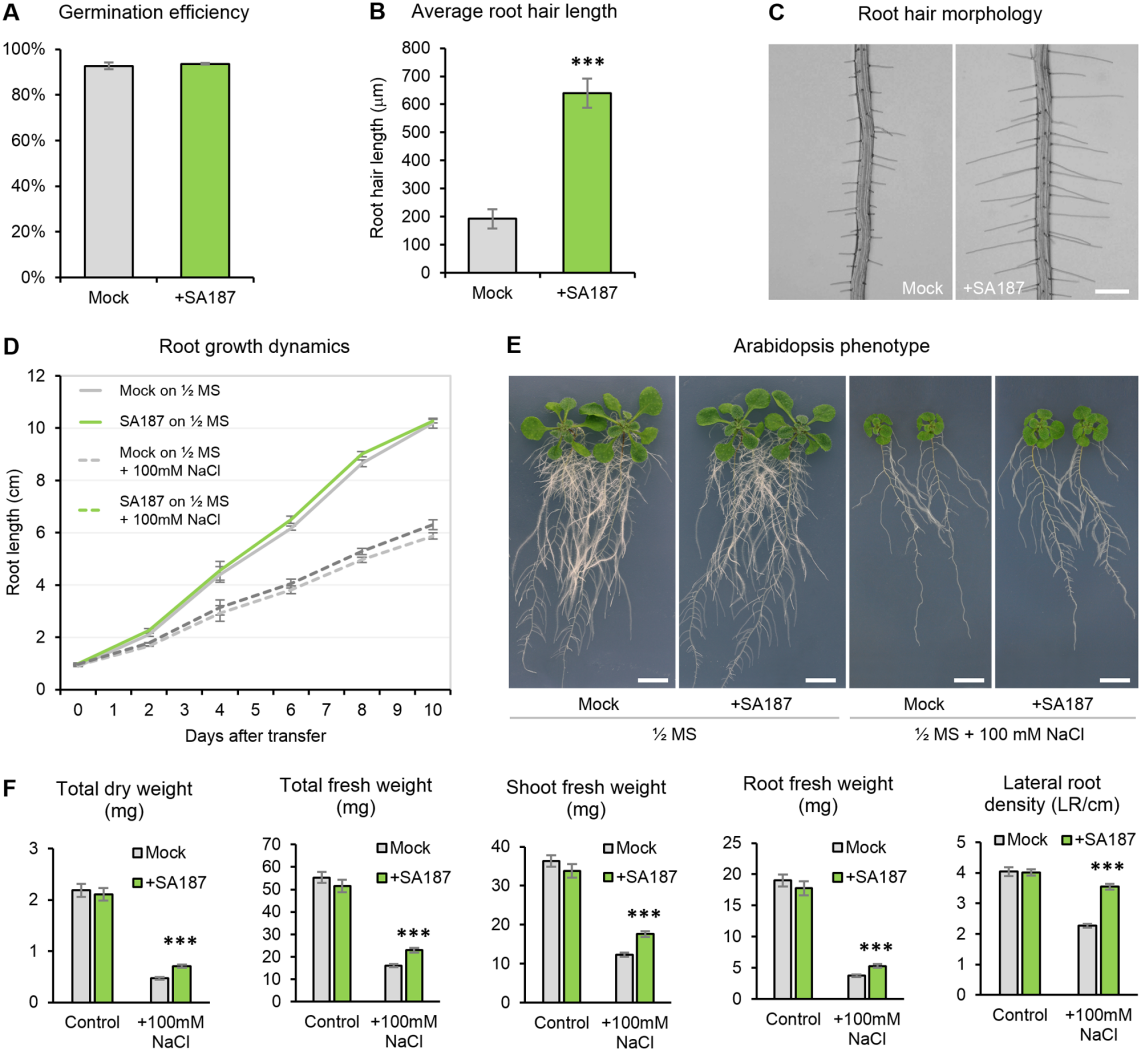
SA187 enhances Arabidopsis tolerance to salt stress. (A) Germination efficiency on ½ MS medium without (Mock) or with SA187 (+SA187) (n > 300, 3 biological replicates, error bars represent SE). (B) Average root hair length of 10% longest root hairs (n > 70) in 5-day-old seedlings grown vertically on ½ MS medium without (Mock) or with SA187. Error bars represent SD. (C) Typical root hair morphology of 5-day-old seedlings used for the analysis in (B). Bar represent 200 μm. (D) Root length time course of SA187-inoculated or mock-inoculated Arabidopsis seedlings after transfer of 5-day-old seedlings from ½ MS to ½ MS with or without 100mM NaCl (n = 60). Error bars represent SE. (E) SA187-colonized 17-day-old plants showing enhanced growth under salt stress (½ MS + 100mM NaCl) but negligible differences under normal conditions (½ MS). Plants were treated as shown in S2 Fig. Bars represent 1 cm. (F) Total plant fresh weight, shoot fresh weight, root fresh weight, total plant dry weight of 17-day-old seedlings and lateral root density of 13-day-old seedlings inoculated by SA187 or mock-treated transferred 5 days after germination from ½ MS to ½ MS with or without 100mM NaCl. All plots represent the mean of 3 biological replicates (n > 39). Error bars represent SE. Asterisks indicate a statistical difference based on the Student’s t-test (* P < 0.05; ** P < 0.01; *** P < 0.001).

To better understand the molecular mechanism by which SA187 confers stress tolerance to plants, we used the genetic model plant *A*. *thaliana* and first assessed the capacity of SA187 to affect the early stages of Arabidopsis development under normal conditions (½ MS agar medium, 22°C, 16 h of light). When compared to mock-inoculated plants, SA187 had no influence on the germination rate of Arabidopsis seeds (Fig 2A), and apart from considerably longer root hairs (Fig 2B and 2C), 5-day-old seedlings showed no morphological changes. Similarly, after transfer onto new ½ MS plates (S2 Fig), no differences between 17-day-old mock- and SA187-inoculated seedlings were recorded, when measuring root length, lateral root density, shoot morphology, or root and shoot fresh and dry weight of seedlings (Fig 2D–2F) indicating that SA187 has no significant effect on Arabidopsis development under normal growth conditions.

On the other hand, the stress tolerance and growth promoting capacity of SA187 on Arabidopsis was highlighted under salt stress. Five days after germination, SA187- and mock-inoculated seedlings were transferred onto ½ MS agar plates supplemented with 100 mM NaCl (S2 Fig), and the same growth parameters as abobe were evaluated up to 12 days after the transfer to salt plates. SA187-inoculated plants showed stress tolerance promoting activity on salt stress: the shoot and root systems of SA187-inoculated plants were significantly more developed than those of mock-inoculated plants (Fig 2E and 2F). While primary root length was similar between SA187- and mock-inoculated plants (Fig 2D), lateral root density was significantly increased (Fig 2F). Similarly to 5-day-old seedlings, SA187-inoculated plants at this stage had more than twice longer root hairs compared to the mock-inoculated ones under both normal and salt stress conditions (S3 Fig). Moreover, we proved that the beneficial activity of SA187 was largely linked to living bacterial cells as heat-inactivated SA187 cells did not induce any beneficial activity (S4A Fig). Overall, SA187 strongly enhanced Arabidopsis growth of both shoot and root under salt stress conditions, in contrast to normal conditions.

### *Enterobacter* sp. SA187 modifies root and shoot K^+^ levels

**Fig 3:**
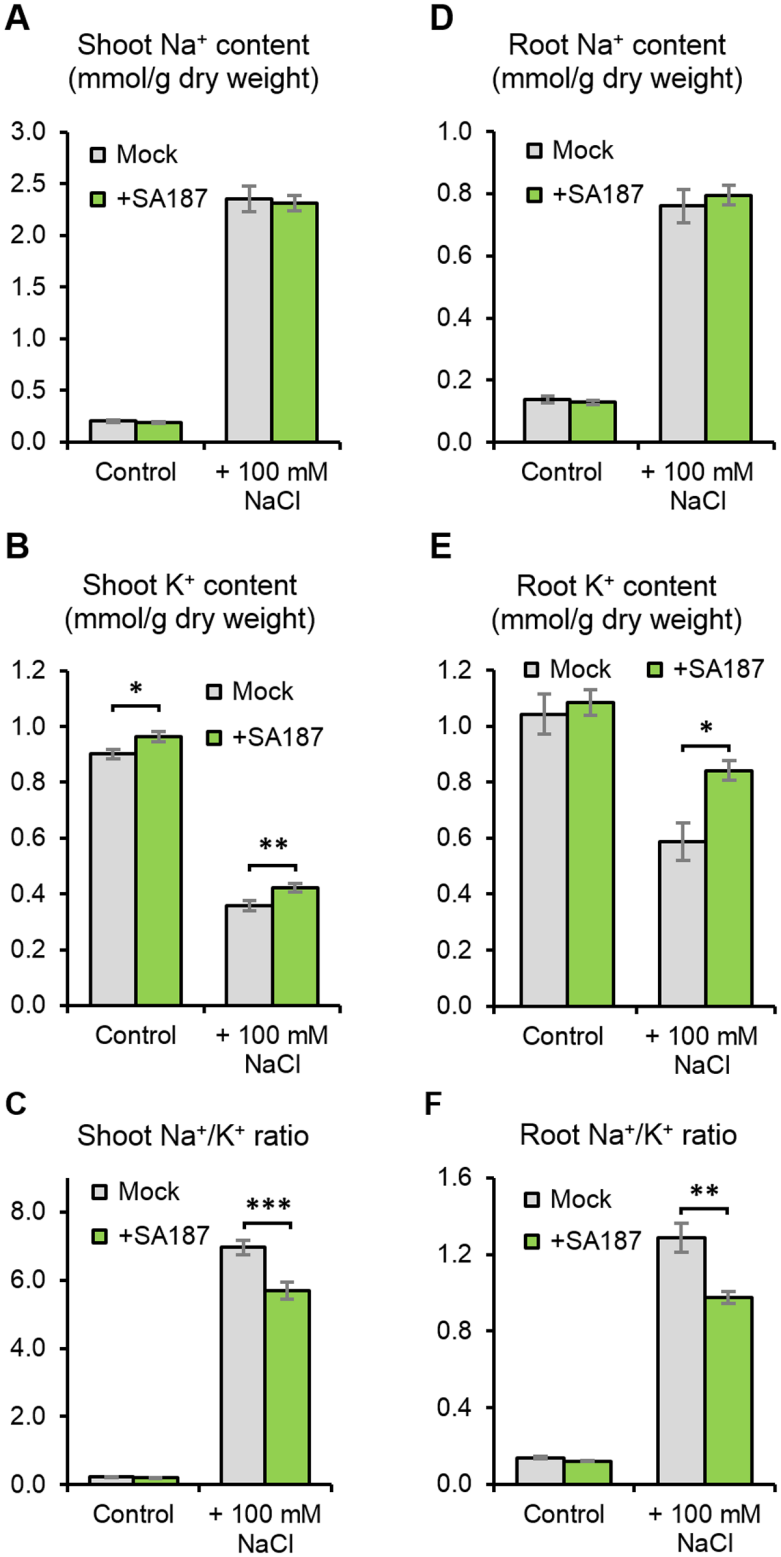
Ion content in Arabidopsis seedlings. Shoot Na^+^ content (A), shoot K^+^ content (B) and shoot Na^+^/K^+^ ratio (C) of 17-day-old mock- or SA187-inoculated Arabidopsis seedlings exposed for 12 days to ½ MS with or without 100 mM NaCl (48 > n > 36). Root Na+ content (D), root K^+^ content (E) and root Na^+^/K^+^ ratio (F) of 17-day-old mock- or SA187-inoculated Arabidopsis seedlings exposed for 12 days to ½ MS with or without 100mM NaCl (48 > n > 12). All plots represent the mean of three biological replicates, and error bars represent SE. Asterisks indicate a statistical difference based on the Mann-Whitney test (* P < 0.05; ** P < 0.01; *** P < 0.001).

The concentration of sodium (Na^+^) and potassium (K^+^) ions in shoots is an important parameter for salt stress tolerance [34]. Therefore, the Na^+^ and K^+^ contents were determined in Arabidopsis organs in the absence and presence of SA187. Interestingly, both shoots and roots of SA187-inoculated plants accumulated similar levels of Na^+^ compared with mock-inoculated plants under normal and salt stress conditions (Fig 3A and 3D). However, increased K^+^ levels were found in SA187-inoculated plants (Fig 3B and 3E), resulting in significantly reduced shoot and root Na^+^/K^+^ ratios under saline conditions (Fig 3C and 3F), which may help the inoculated plants to keep high growth rate.

### *Enterobacter* sp. SA187 colonizes epidermis and inner tissues of both roots and shoots

**Fig 4:**
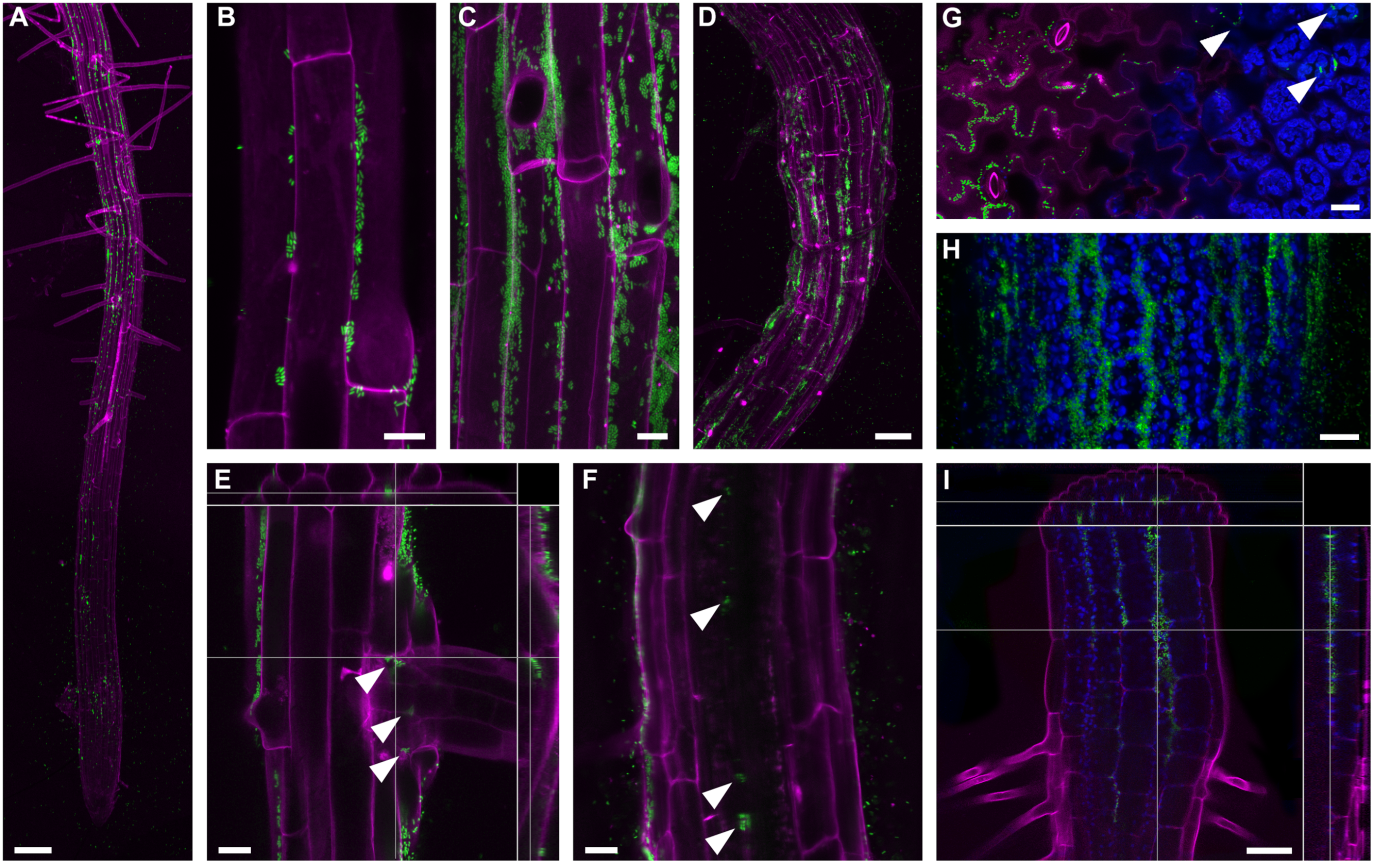
Colonization of Arabidopsis seedlings with GFP-expressing SA187 visualized by confocal microscopy. (A) Root colonization of agar-grown seedlings starts in the elongation zone. Large colonies then occur in the differentiation zone. MIP; bar = 100 μm. (B) Colonies first established themselves in grooves between root epidermal cells. MIP; bar = 10 μm. (C) Large colonies in the differentiation zone grow out from the grooves. MIP; bar = 10 μm. (D) Root colonization of soil-grown seedlings exhibit a more random pattern in comparison to agar-grown seedlings. MIP; bar = 50 μm. (E) Lateral root emergence allows SA187 to enter the root and colonize the lateral root base (marked by arrowheads). A selected confocal section from a Z-stack with top and side orthogonal views. Bar = 20 μm. (F) Scattered SA187 colonies occur inside the root tissues in two-week-old seedlings (marked by arrowheads). A single confocal section. Bar = 20 μm. (G) In cotyledons, SA187 colonizes grooves between epidermal cells (left side) as well as the extracellular space between mesophyll cells (right side; marked by arrowheads). A single oblique confocal section is shown. Bar = 20 μm. (H) SA187 colonization of the hypocotyl epidermis. MIP; bar = 20 μm. (I) SA187 cells enter hypocotyl via stomata, move freely among hypocotyl cells and occasionally establish colonies inside. A selected confocal section from a Z-stack with top and side orthogonal views. Bar = 50 μm. Green–SA187-GFP; Magenta–cells walls (propidium iodide labeling); Blue–chloroplasts (autofluorescence); MIP–maximum intensity projection of a confocal Z-stack.

After recognition of the beneficial impact of SA187 on plant physiology, we wanted to characterize the interaction of SA187 with plants in more detail, and find whether SA187 is able to efficiently colonize Arabidopsis as its non-native host. SA187 cells were stably transformed to express GFP (SA187-GFP), which did not affect their beneficial effect on Arabidopsis seedlings (S4B Fig). Confocal microscopy revealed that SA187-GFP colonized both roots and shoots on ½ MS agar plates or in soil (Fig 4). On vertical ½ MS agar plates, the first colonies (formed by a small number of cells) were observed on the root epidermis in the elongation zone, preferentially in grooves between epidermal cell files (Fig 4A and 4B). In the differentiation zone and older root parts, colonies were larger and proportional with the age of the region (Fig 4C). A similar colonization pattern was observed in soil-grown seedlings, however, with a more random distribution of colonies (Fig 4D). SA187-GFP colonies were also often found in cavities around the base of lateral roots (Fig 4E). While it was rare to detect SA187-GFP cells inside root tissues in 5–7 days old seedlings, the apoplast of the root cortex and even of the central cylinder was regularly occupied by small scattered colonies in 3 weeks old seedlings (Fig 4F). Indeed, in our initial plant assays, SA187 could be re-isolated from surface sterilized Arabidopsis roots, indicating that SA187 was proliferating inside root tissues. Inspecting shoots, SA187-GFP colonies were found deep inside the apoplast of hypocotyls, cotyledons and the first true leaves, and in several cases, bacterial cells were directly observed to penetrate through stomata of these organs (Fig 4G–4I).

Furthermore, we evaluated colonization of root systems by SA187 (wild type strain) under normal and salt conditions. Plants were germinated on ½ MS agar plates containing SA187 wild type strains, transferred to new ½ MS plates with or without 100 mM NaCl after 5 days (S2 Fig), and parts of their root systems grown after the transfer were used for bacterial extraction after 5 more days. Interestingly, quantification based on counting of colony forming units (CFU) revealed that roots from salt conditions were twice more colonized than those from normal conditions (S5 Fig), suggesting that in our experimental system plants can probably facilitate their accessibility to colonization by beneficial bacteria under stress conditions.

### SA187 massively reprograms Arabidopsis gene expression upon colonization

**Fig 5:**
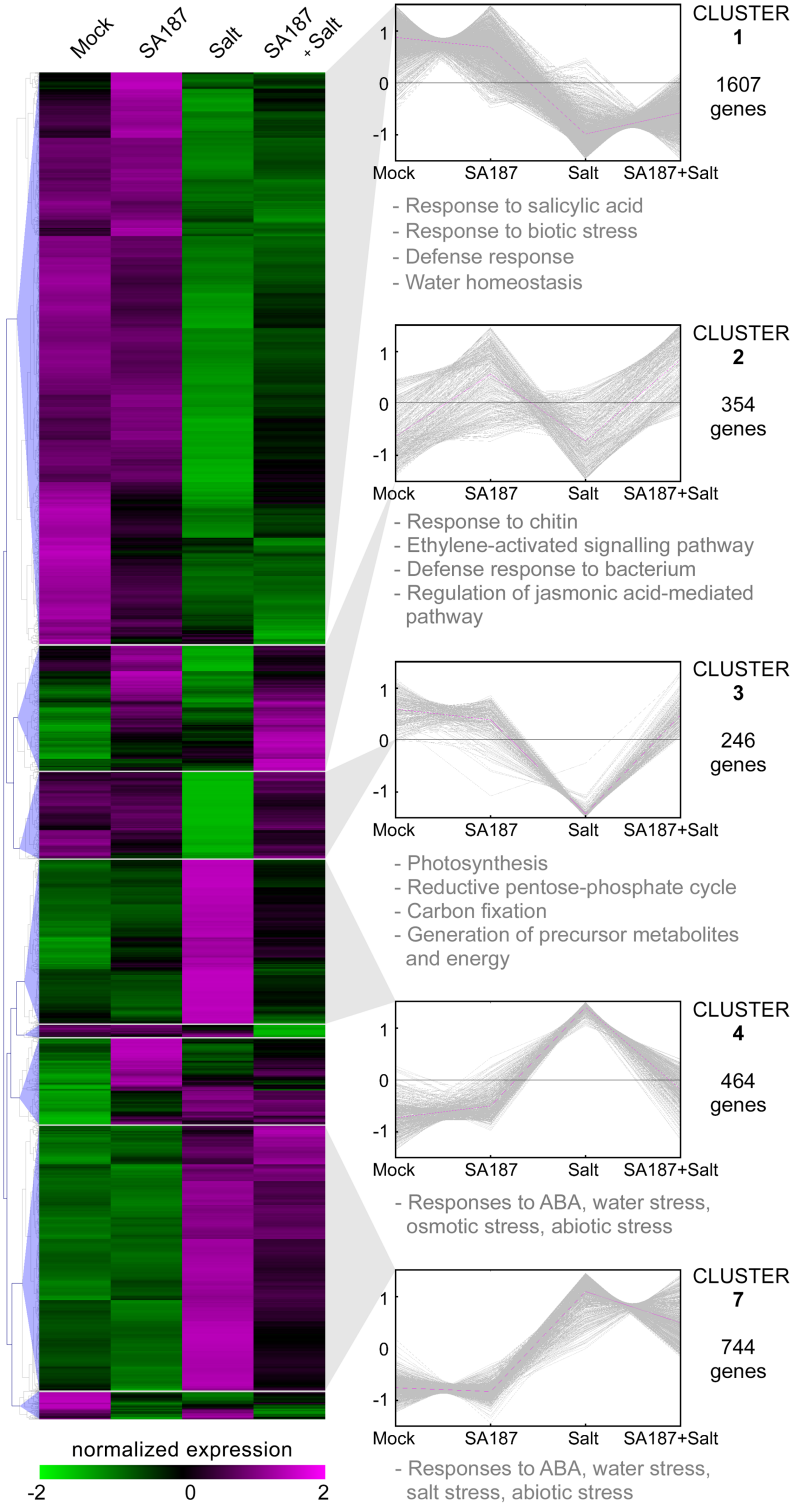
Transcriptome analysis of Arabidopsis response to SA187. Hierarchical clustering of up- and down-regulated genes in Arabidopsis seedlings in response to SA187, salt (100 mM NaCl) or both treatments based on the RNA-Seq analysis. For every gene, FPKM values were normalized. Heat map colors indicate expression levels. For the most relevant clusters, gene families significantly enriched are indicated based on gene ontology.

To uncover how salt stress tolerance is achieved in SA187-inoculated Arabidopsis seedlings, we performed RNA-Seq analysis comparing the transcriptome of mock-inoculated to SA187-inoculated plants under non-saline (Mock, SA187), and salt stress conditions (Salt, SA187+Salt). Compared to “Mock” conditions, 545, 3113 and 1822 genes were found to be differentially expressed in the “SA187”, “Salt” and “SA187+Salt” samples, respectively (S1 Table). To obtain a global overview, the transcriptome data were organized by hierarchical clustering into 8 groups and analyzed for gene ontology enrichment (Fig 5, S2 Table).

Cluster 1 and 7 comprise the largest sets of differentially expressed genes with 1607 and 744 members, respectively, and consist of salt-stress regulated genes that were unaffected by the SA187 inoculation. Whereas Cluster 1 genes are strongly downregulated under salinity and are involved in water homeostasis, salicylic acid (SA) and defense response, those of Cluster 7 are highly upregulated and enriched in genes that are induced in response to water and salt stress or abscisic acid (ABA).

A specific effect of SA187 on the transcriptome of plants was found in Clusters 2, 3 and 4. Cluster 2 (354 genes) represents genes that are upregulated by SA187 independently of the growth conditions. This cluster is significantly enriched in plant defense genes such as chitin responsive genes but also in ethylene and jasmonic acid (JA) signaling (Fig 5). Importantly, Cluster 3 genes (246) are strongly downregulated in mock-inoculated plants under salt stress conditions but remain unaltered upon SA187-inoculation. These genes have a role in the primary metabolism, such as photosynthesis, carbon and energy metabolisms. On the contrary, Cluster 4 genes (464) are enriched in ABA and abiotic stress response and are upregulated in salt-treated plants, but not when the plants were inoculated with SA187.

In summary, these data indicate that SA187 colonization triggers in Arabidopsis the expression of genes involved in defense response as shown by the significant enrichment for chitin responsive genes and ethylene and JA signaling. Moreover, under saline conditions, SA187-inoculated plants release themselves from the impact of abiotic stress (ABA), maintain higher metabolic and photosynthetic activity, and can therefore grow better than mock-inoculated plants.

### SA187 modulates abscisic acid, jasmonic acid, and ethylene hormonal pathways under salt stress

**Fig 6:**
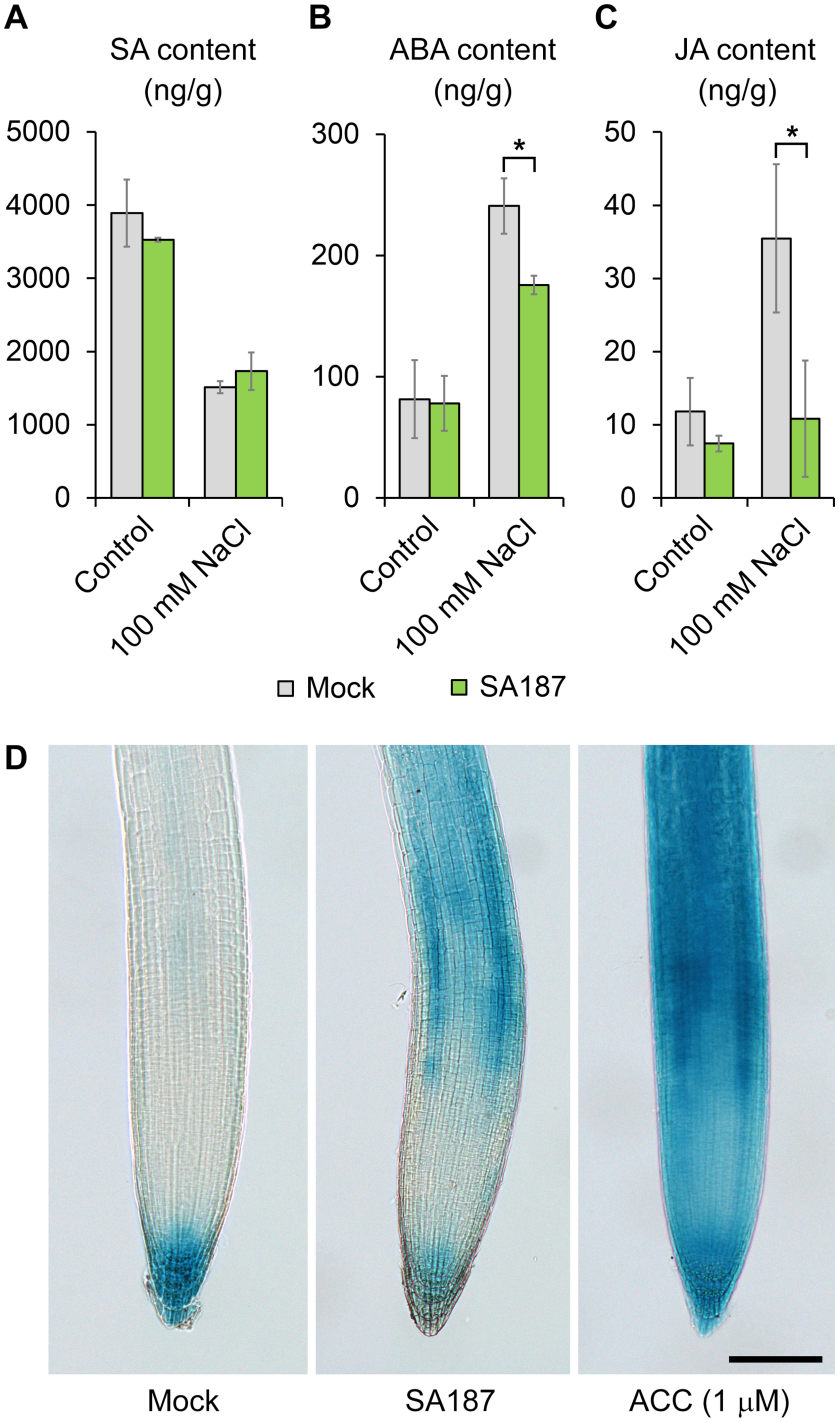
SA187 modulates abscisic acid, jasmonic acid, and ethylene hormonal pathways under salt stress. (A) Salicylic acid (SA), (B) abscisic acid (ABA) and (C) jasmonic acid (JA) content in mock- and SA187-inoculated plants after growth on ½ MS with or without 100 mM NaCl for 12 days. Error bars indicate SE, based on three biological replicates. Asterisks indicate a statistical difference based on the Mann-Whitney test (* P < 0.05). (D) The ethylene reporter, *pEBF2*::*GUS*, visualizing the relative ethylene content in primary root tips of mock- and SA187-inoculated, and ACC-treated 7-day-old seedlings under normal conditions (salt stress conditions provided similar results). Bar = 100 μm.

Since our transcriptome analysis indicated possible roles of several hormone pathways in the SA187-induced growth promotion under salt stress, we measured the levels of salicylic acid (SA), jasmonic acid (JA) and abscisic acid (ABA) in mock- and SA187-inoculated plants. SA187 did not significantly change plant SA levels in the absence or presence of salt (Fig 6A). Plant ABA and JA concentrations remained also unchanged upon SA187 colonization under normal conditions, but their salt-induced accumulation was significantly lower in SA187-inoculated plants (Fig 6B and 6C), indicating a partial attenuation of stress responses in these plants.

To assess the level of ethylene in Arabidopsis roots and possibly confirm the activation of the ethylene signaling pathway observed in Cluster 2, we used the ethylene-dependent *pEBF2*::*GUS* reporter [35]. In contrast to mock-inoculated seedlings, the reporter line showed strong GUS activity in root tips upon SA187-inoculation, similar to the treatment with the ethylene precursor aminocyclopropane-1-carboxylic acid (ACC) (Fig 6D), indicating the activation of the ethylene signaling pathway.

### Ethylene perception mutants are compromised in the beneficial response to SA187

**Fig 7:**
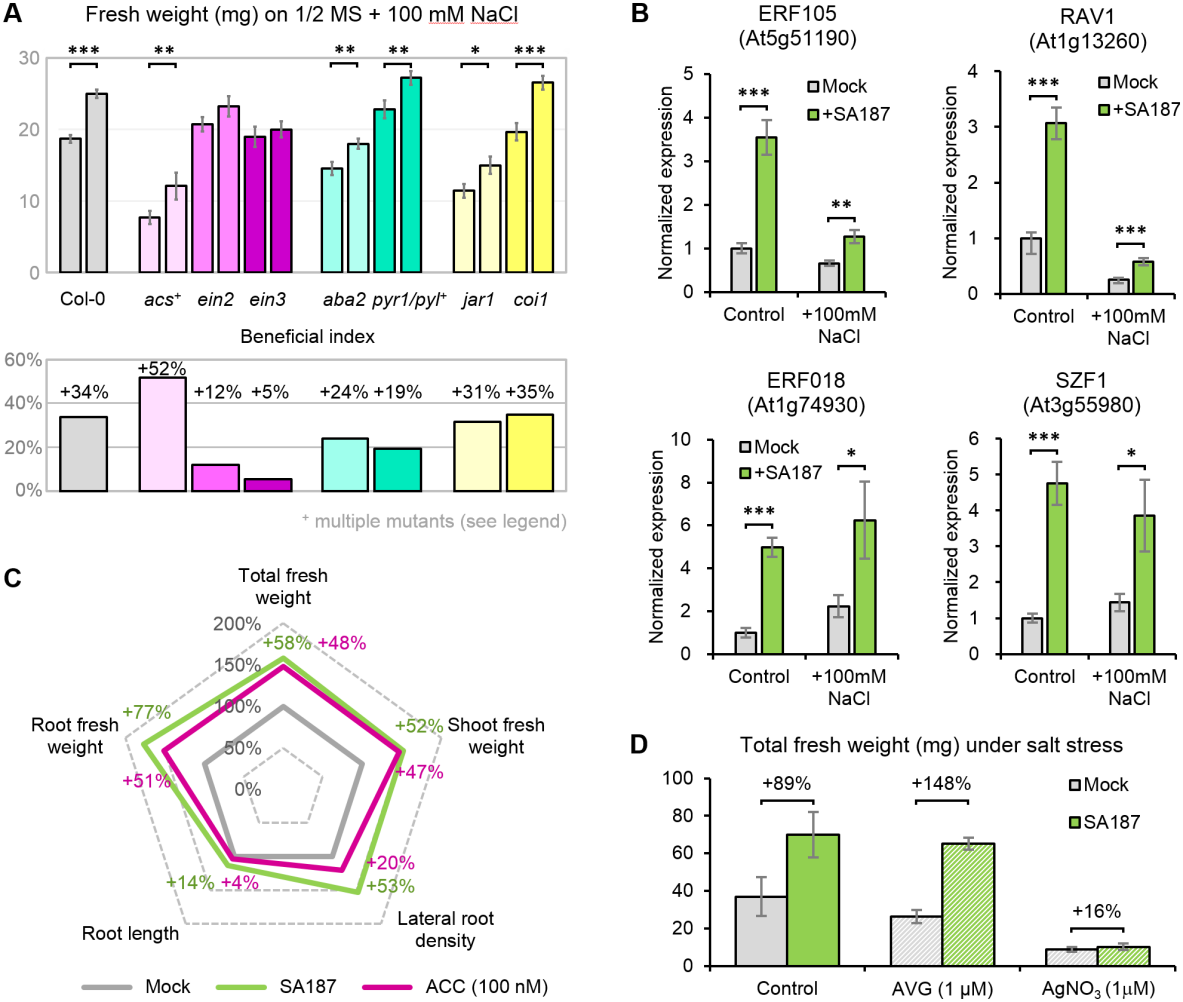
Ethylene signaling is important for the beneficial effect of SA187 under salt stress. (A) Fresh weight and beneficial index (a ratio between fresh weight of SA187- and mock-inoculated seedlings) of mutants in hormonal pathways transferred from ½ MS to ½ MS + 100 mM NaCl (5+12 days). *acs* = heptuple mutant *acs1-1 acs2-1 acs4-1 acs5-2 acs6-1 acs7-1 acs9-1*, and *pyr1/pyl* = quadruple mutant *pyr1 pyl1 pyl2 pyl4*. All plots represent the mean of three biological replicates (n > 36). Error bars represent SE. (B) qPCR expression analysis of four ethylene-associated genes in 17-day-old mock- and SA187-inoculated Arabidopsis seedlings exposed for 12 days to ½ MS with or without 100 mM NaCl. Normalized expression indicates the linear fold change compared to mock-treated plants on ½ MS. Values represent means of three biological experiments, each in three technical replicates. Error bars indicate SE. (C) 100 nM ACC partially mimics the effect of SA187 on salt stress tolerance improvement in Arabidopsis seedlings. Five-day-old-seedlings were transferred to ½ MS + 100 mM NaCl with or without ACC and evaluated after 12 days. SA187-inoculated plants were used for comparison. (D) Total fresh weight of mock- and SA187-inoculated 18-day-old Arabidopsis seedlings on ½ MS with 100 mM NaCl supplemented with the ethylene synthesis inhibitor AVG or ethylene signaling inhibitor AgNO_3_. Error bars representing SE and beneficial index (%) are displayed. Asterisks indicate a statistical difference based on the Student’s t-test (* P < 0.05; ** P < 0.01; *** P < 0.001).

To substantiate the phytohormone quantifications, Arabidopsis hormone deficient or insensitive mutants were analyzed. The JA-receptor *coi1-1* mutant [36], the JA-insensitive *jar1-1* mutant [37], the ABA biosynthesis *aba2-1* mutant [38] or the ABA receptor quadruple mutant *pyr1-1 pyl1-1 pyl2-1 pyl4-1* (named here as *pyr1/pyl*) [39] maintained the SA187 beneficial activity upon salt stress, indicating that ABA or JA may not play a major role in this interaction (Fig 7A, S6 Fig).

However, the ethylene insensitive *ein2-1* and *ein3-1* mutants [40,41], impaired in ethylene perception, were strongly compromised in the beneficial effect of SA187, indicating that ethylene sensing could be of importance in SA187-induced tolerance of Arabidopsis to salt stress conditions. This result was confirmed by the up-regulation of the four ethylene-induced genes, *ERF106*, *ERF018*, *RAV1* and *SZF1*, upon colonization by SA187 (Fig 7B). Moreover, application of 100 nM ACC during salt stress could largely mimic the beneficial activity of SA187 on plants (Fig 7C, S7 Fig).

In contrast, the heptuple ethylene-biosynthesis deficient mutant *acs1-1 acs2-1 acs4-1 acs5-2 acs6-1 acs7-1 acs9-1* (called *acs* in this study) still showed full sensitivity to the beneficial activity of SA187 under salt stress (Fig 7A). Additionally, the SA187 beneficial effect was maintained when plants were treated with amino-ethoxy-vinyl glycine (AVG, 1 μM), an ethylene production inhibitor blocking ACC synthesis [42] (Fig 7D). However, when plants were treated with silver nitrate (AgNO_3_, 1 μM), which interferes with ethylene perception [42], SA187-inoculated plants did not exhibit any SA187-induced tolerance to salt stress (Fig 7D).

Altogether, these results indicate that the beneficial effect of SA187 may not be mediated by JA perception or the ABA pathway, but rather by the ethylene perception, as it was found to be necessary for SA187-induced salt stress tolerance on Arabidopsis plants.

### Arabidopsis upregulates the methionine salvage pathway in SA187

**Fig 8:**
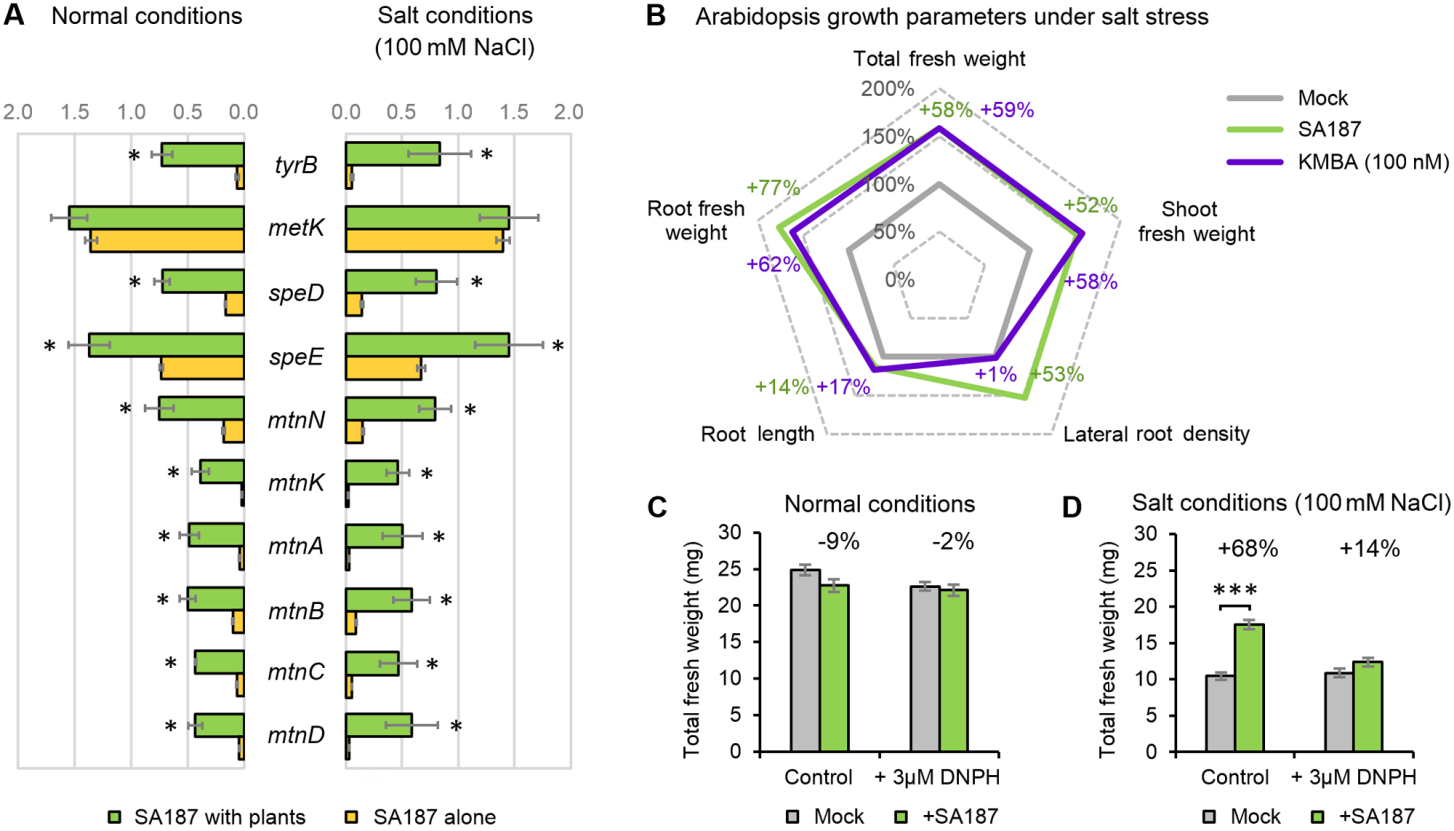
KMBA as a potential ethylene precursor in the plant-SA187 interaction. (A) qPCR analysis of the methionine salvage pathway gene expression of SA187 colonizing plants in control or salt stress conditions compared to SA187 cultivated alone in ½ MS with or without 100 mM NaCl. Values represent means of three biological experiments, each in three technical replicates. Error bars indicate SE. (B) KMBA partially mimics the effect of SA187 on salt stress tolerance improvement in Arabidopsis seedlings. Plants were transferred 5 days after germination to ½ MS + 100 mM NaCl with or without KMBA and evaluated after 12 days. SA187-inoculated plants transferred to ½ MS + 100 mM NaCl were used as a positive control. (C, D) Total fresh weight of mock- and SA187-inoculated 17-day-old Arabidopsis seedlings grown on ½ MS medium (C) or ½ MS with 100 mM NaCl (D) supplemented with 3 μM DNPH. All plots represent the mean of four biological replicates (n > 75). Error bars representing SE, beneficial index (%) is displayed. Asterisks indicate a statistical difference based on the Student’s t-test (*** P < 0.001).

The previous results suggested that ethylene most likely originates from SA187 cells rather than from the canonical plant ACC synthase (ACS) pathway. To support the hypothesis that SA187 provides ethylene to promote plant growth, we searched for bacterial genes encoding ACS or ethylene forming enzymes (EFE) [43] in the genome of SA187 [31]. No ACS- or EFE-related genes were found in SA187, which on the other hand, contains a conserved methionine salvage pathway (also known as 5’-methyl-thioadenosine cycle), and one of its components, KMBA, is known to be an ethylene precursor [44]. While SA187 alone did not produce ethylene when grown on synthetic media (S8 Fig), the expression level of most of the genes encoding proteins involved in the methionine salvage pathway were upregulated in SA187 upon plant colonization compared with bacteria incubated for 4h in liquid ½ MS with or without 100 mM NaCl in the absence of plants (Fig 8A).

To confirm that KMBA could function as an ethylene precursor during the beneficial plant-microbe interaction, we tested the effect of KMBA on Arabidopsis in comparison to SA187 inoculation. Under salt stress conditions, application of 100 nM KMBA induced a similar beneficial activity on Arabidopsis as SA187 resulting in a similar increase in both root and shoot fresh weight (Fig 8B, S7 Fig).

Finally, we took the advantage of 2,4-dinitrophenylhydrazine (DNPH), a known interactor of KMBA *in vitro* that was previously shown to precipitate KMBA produced by *Botrytis cinerea* and consequently impairs the production of ethylene by photo-oxidation [33]. Here, we could show that when plants were cultivated with 3 μM DNPH, the beneficial impact of SA187 on Arabidopsis growth under salt stress was greatly reduced from 68% to 14% (Fig 8C and 8D), showing the importance of KMBA in mediating SA187-induced plant tolerance to salt stress.

## Discussion

*Enterobacter* sp. SA187 was previously isolated from the desert pioneer plant *Indigofera argentea* Burm.f. (Fabaceae) [29,31]. In this work, we show that this bacterium promotes plant tolerance to salt stress, describing this strain as a stress tolerance-promoting bacterium. Indeed, under field conditions, using SA187 as an inoculum for alfalfa seeds and by monitoring growth parameters and yield over two different agriculture seasons, the inoculated plants showed a clear improvement in yield independently of the water regime applied (high or low salt stress). The data show similar effectiveness of the SA187 inoculations in both years. However, the differences for high and low-saline conditions were reduced during the second year (Fig 1), which could be explained by the increased rainfall (S1 Fig) during the 2^nd^ growing season that may have diluted the salinity effects. We conclude that SA187 can efficiently improve crop productivity under extreme agricultural conditions and could be a simple biological solution to grow plants under extreme adverse conditions.

In order to understand the mechanisms underlying the beneficial plant interaction with SA187, Arabidopsis was used as a model system. SA187 colonizes both surface and inner tissues of Arabidopsis roots and shoots, supporting a functional plant-bacterial interaction (Fig 4). Colonization of both above- and under-ground organs is in agreement with the observation that leaf and root microbial communities share an important portion of their bacterial species [11]. While the mechanism of entry of SA187 into roots occurs most probably via cracks and/or by active penetration between epidermal cells [45], we observed that shoots were colonized through stomata, indicating that these apertures represent a major route of entry into plants not only by pathogenic but also by beneficial bacteria.

The capacity of SA187 to enhance salt stress tolerance of Arabidopsis was analyzed in detail. While SA187 induced only negligible morphological and physiological changes in plants under non-stress conditions (with the exception of longer root hairs), SA187 significantly enhanced root and shoot growth with increased fresh and dry weight under salt stress (Fig 2E and 2F). In addition, SA187 increased lateral root density, and thus the overall root surface area (Fig 2F) under salt stress. Changes in the root architecture have been considered to be beneficial for adaptation to various abiotic stress conditions including salinity [46], and very likely contribute to the SA187-induced salt tolerance in Arabidopsis.

The effect of salinity on plants includes two components: an osmotic component, being the consequence of an altered osmotic pressure due to an increased salt concentration, and a toxic ion effect as a result of the high Na^+^ concentration in shoots [47,48]. The toxic effects of the Na^+^ accumulation result in premature senescence, leading to a decrease in photosynthesis efficiency and impaired metabolic processes. Na^+^ also competes with K^+^ in membrane transport and enzymatic functions, reducing plant growth. Most plant cells possess mechanisms to counteract the harmful effects of Na^+^ accumulation by retaining K^+^ and actively excluding Na^+^ in roots and/or sequestering Na^+^ in vacuoles in shoots [47–50]. Several studies have shown that an inoculation of commercial crops, such as maize, strawberry and wheat by PGPBs under salt stress results in a decrease of Na^+^ and an increase of K^+^ in their shoots and leaves [51–53]. The inoculation of *Arabidopsis thaliana* and *Trifolium repens* (white clover) by *Bacillus subtilis* GB03 induced a decrease in the Na^+^ content in shoots in both species accompanied by an increase or no change in the K^+^ content [54,55]. In our study, we found no differences in Na^+^ contents in shoots or roots between SA187-inoculated and mock-inoculated plants in response to salt stress. However, K^+^ ion levels in both roots and, to a lesser extent, shoots increased upon the SA187 inoculation, resulting in reduced Na^+^/K^+^ ratios (Fig 3C and 3F), which might contribute to the higher salt tolerance of SA187-inoculated plants [56].

To analyze the interaction of SA187 with Arabidopsis at the molecular level, the transcriptome of Arabidopsis grown under salt and non-stress conditions in the absence or presence of SA187 was compared. The inoculation with SA187 dramatically reprogrammed the gene expression of plants grown either on ½ MS or on ½ MS with 100 mM NaCl. This was highlighted in Clusters 2, 3, and 4 of the RNA-Seq analysis (Fig 5). Cluster 3 genes, mostly related to photosynthesis and primary metabolism, were strongly downregulated under salt stress in mock-inoculated plants, confirming previously published reports which correlated such a downregulation with the inhibition of growth and development under salt stress conditions [57]. These results could therefore explain why SA187-inoculated plants grow better under stress conditions: SA187-inoculated plants only mildly reduce their photosynthetic capacity and maintain a functional metabolism allowing further growth in comparison to mock-inoculated plants. Cluster 4 genes are enriched in ABA-related stress genes and were induced upon salt stress in mock-inoculated plants, but not in SA187-inoculated plants. These results indicate that some salt stress-induced responses, including the enhancement of ABA levels, are dampened by SA187. However, they do not explain why plants are more salt stress tolerant. Indeed, the ABA biosynthesis *aba2-1* mutant or the ABA receptor quadruple mutant *pyr1-1 pyl1-1 pyl2-1 pyl4-1* still exhibited a similar growth improvement by SA187 as wild-type plants when exposed to salt stress, indicating that ABA production and signaling are dispensable in the presence of these beneficial bacteria (Fig 7A).

Induced salt stress tolerance by SA187 could be elucidated by Cluster 2, comprising genes specifically induced upon SA187-inoculation. This cluster is significantly enriched for genes involved in defense response to bacterium, and for chitin response. This latter GO term is not surprising in a plant-bacterial system, since pathogen associated molecular patterns (PAMPs) such as fungal chitin and bacterial flagellin are inducing a large set of common genes in plants, with more than 60% of overlap [58]. But the most interesting feature lies in the enrichment of the ethylene response pathway. Indeed, SA187 activates the ethylene perception pathway as shown by the qPCR analysis of ethylene-induced genes and by the ethylene reporter *pEBF2*::*GUS* (Figs 6D and 7B). Moreover, ACC and KMBA as ethylene precursors largely mimicked the beneficial effect of SA187 on plants under salt stress (Figs 7C and 8B). Finally, the involvement of ethylene was also supported by the observation of much longer root hairs (Fig 1B and 1C; S3 Fig), as ethylene plays an important role in root hair elongation [59,60]. Although the role of ethylene in plant abiotic stress tolerance is controversial [61], several pieces of evidence indicate that this phytohormone is important for plant adaptation to abiotic stresses. For example, the pre-treatment of Arabidopsis seedlings with ACC, or the use of the constitutive ethylene response (*CTR1*) or the *EIN3* gain-of-function mutants were shown to enhance salt stress tolerance [62,63]. Furthermore, an ethylene overproduction in the *eto1* mutant lead to salinity tolerance due to improved Na^+^/K^+^ homeostasis through an RBOHF-dependent regulation of Na^+^ accumulation [64].

Importantly, ethylene-related Arabidopsis mutants revealed that the beneficial activity of SA187 is to a major extent mediated via the perception of externally produced ethylene. Although the *ein2-1* and *ein3-1* mutants were compromised in their beneficial response to SA187, the disruption of the plant ethylene production in the heptuple *acs* mutant showed the same growth enhancement under salt stress when comparing SA187-inoculated plants to mock-inoculated plants (Fig 7A). This was supported by a parallel pharmacological approach, demonstrating that inhibition of the ACS activity using AVG did not block the stress tolerance promoting activity of SA187, while blocking the ethylene receptors by AgNO_3_ compromised the beneficial activity of SA187 on plants under salt stress (Fig 7D).

As plants were shown to perceive ethylene even without functional plant ethylene production, we suspect that SA187 could provide plants with ethylene or its precursor. Three main pathways for ethylene biosynthesis have been described in bacteria and other microbes. The mold *Dictyostelium mucoroides* and fungi *Penicillium citrinum* produce ethylene from methionine via S-adenosyl-methionine, through the sequential action of ACC synthases and ACC oxidases. S-adenosyl-methionine is first converted to ACC by ACC synthases, which is then oxidized by ACC oxidases to release ethylene and cyanide. The same pathway is well known to be responsible for ethylene biosynthesis in plants, where cyanide is converted to β-cyanoalanine to avoid toxicity [44,65]. Microbes can also produce ethylene from α-ketoglutarate and arginine by the action of the ethylene forming enzyme (EFE), which has been found in several microbial species such as *Pseudomonas syringae* and *Penicillium digitatum* [44,66]. A third pathway has been identified in a variety of bacteria such as *Escherichia coli* and *Cryptococcus albidus*, or in fungi like truffle or pathogenic *Botrytis cinerea*, where ethylene is produced via oxidation of KMBA, an intermediate of the methionine salvage pathway [32,33,44,67]. KMBA can be spontaneously converted to ethylene by photo-oxidation or through the action of peroxidases [33], which are abundantly present in the plant apoplast [68,69].

Based on P-BLAST homology searches, genome analysis of SA187 revealed that neither ACC synthase nor EFE genes are present in SA187. Instead, SA187 contains the entire methionine salvage pathway, suggesting that KMBA is most likely the precursor of ethylene in SA187. Interestingly, most of the methionine salvage pathway genes in SA187 are only actively expressed upon colonization of Arabidopsis (Fig 8A). Moreover, the application of KMBA could mimic the beneficial effect of SA187 on plants when subjected to salt stress (Fig 8B). Importantly, the SA187 beneficial activity towards plant was highly reduced when treated with DNPH, known to provoke KMBA precipitation and prevent thus its oxidation and ethylene release [33].

Taken together, the KMBA involvement in abiotic stress tolerance constitutes a novel mechanism in the field of plant-beneficial bacteria interaction. While the induction of the ethylene signaling pathway by PGPB has been reported in several studies to play an important role in the induced systemic resistance in plants [18,70,71], PGPB activity in the context of abiotic stress has been commonly attributed rather to a reduction of the plant ethylene level through the activity of bacterial ACC deaminases [53,72–74], or shown to be independent of the ethylene signaling pathway [75,76]. Several reports hypothesized the involvement of ethylene signaling in abiotic stress tolerance induced by rhizosphere bacteria, with evidences that were largely based on emissions of unidentified volatiles or by comparison with plant-fungal interactions [32,77,78]. Recently, it has been reported that the beneficial bacterium *Burkholderia phytofirmans* PsJN enhanced plant growth through an auxin/ethylene-dependent signaling pathway under optimal conditions, but in contrast to the present study, the authors hypothesized that the plant intrinsic ethylene production was fundamental in that interaction [79].

In conclusion, we provide evidence that the endophytic bacterium *Enterobacter* sp. SA187 induces salt stress tolerance in Arabidopsis via production of KMBA to activate the ethylene pathway. SA187 enhances plant salt stress tolerance under controlled conditions in the model plant *Arabidopsis thaliana* and under field conditions in the crop plant alfalfa. These results show the potential use of SA187 for bringing saline agriculture of current crops a step closer to reality.

## Methods

### Field trials

To inoculate alfalfa (*Medicago sativa* var. CUF 101) seeds, a slurry was prepared consisting of sterilized peat, a broth culture of SA187, and sterilized sugar solution (10%) in the ratio 5:4:1 (w/v/v). Subsequently, alfalfa seeds were coated with the slurry at a rate of 50 mL·kg^-1^. As a control, seeds were coated with a similar mixture without bacteria. Field trial was conducted at the experimental station in Hada Al-Sham (N 21°47'47.1" E 39°43'48.8"), Saudi Arabia, in winter seasons 2015–2016 and 2016–2017. The experiment was a randomized complete block design with a split-split plot arrangement of four replicates in the for season 2015–2016 season and three replicates in the 2016–2017 season, plots (2 × 1.5 m) with seed spacing 20 cm row-to-row. The field was irrigated using groundwater with two different salinity levels: low salinity (EC = 3.12 dS·m^-1^), and high salinity (EC = 7.81 dS·m^-1^). The soil had an average pH 7.74 and salinity EC = 1.95 dS·m^-1^. Agronomical data (plant height, fresh biomass, and dry biomass) were recorded every 25–30 days from each harvest; three harvests were done in the first season, four harvests in the second season. Field trials data were analyzed as a randomized complete block design using a Factorial ANOVA Model, followed by least significant difference (LSD) test for pairwise comparisons. Results with a p-value < 0.05 were considered significant. All statistical analysis was carried out using SAS/STAT software (https://www.sas.com/).

### Endophytic bacteria, plant material, growth condition and physiological experiments

*Enterobacter sp*. SA187 was previously isolated from root nodules of the leguminous pioneer plant *Indigofera argentea* in the Jizan region of Saudi Arabia [29,31]. Arabidopsis seeds were obtained from publicly available collections. The following mutant lines used in this study were published previously: the JA-receptor *coi1-1* mutant [36], JA-insensitive *jar1-1* [37], the ABA biosynthesis *aba2-1* mutant [38], the ABA receptor quadruple *pyr1-1pyl1-1pyl2-1pyl4-1* mutant [39], the ethylene insensitive *ein2-1* [40] and *ein3-1* mutants [41], the heptuple ethylene-biosynthesis deficient mutant *acs1-1acs2-1acs4-1acs5-2acs6-1acs7-1acs9-1* [80], and the ethylene-dependent *pEBF2*::*GUS* reporter [35].

Prior to every experiment, *A*. *thaliana* seeds were surface sterilized 10 min in 70% ethanol + 0.05% sodium dodecyl sulfate on a shaker, washed 2 times in 96% ethanol and let to dry. To ensure SA187-inoculation, sterilized seeds were sown on ½ MS plates (Murashige and Skoog basal salts, Sigma) containing SA187 (2·10^5^ cfu·ml^-1^), stratified for 2 days at 4°C in the dark and then placed vertically to growth conditions for 5 days as shown as in S2 Fig. The ½ MS plates with SA187 were prepared by addition of 10^7^ bacteria to 50 ml pre-cooled agar medium during plate preparation.

Average length of root hairs was determined based on images of 5-day-old roots (1 image per root at constant distance from the root tip, 25 seedlings per condition) or 16-day-old roots (along the whole primary root length grown after transfer) captured by a Nikon AZ100M microscope equipped with an AZ Plan Apo 2x objective and a DS-Ri1 camera (Nikon). All root hairs in focus were measured using ImageJ (https://imagej.nih.gov/ij/). Average values and standard deviations were calculated from 10% longest root hairs to eliminate non-developed root hairs and describe the maximal elongation capacity of root hairs.

For salt stress tolerance assays, 5-day-old seedlings were transferred onto ½ MS plates with or without 100 mM NaCl (Sigma). Primary root length was measured every 2 days using ImageJ software after scanning the plates. Lateral root density was evaluated as detectable number of lateral roots under a stereo microscope divided by the primary root length. Fresh weight of shoots and roots was measured 12 days after transfer of seedlings. Dry weight was measured after drying shoot and shoots for 2 days at 70°C. Following Koch’s postulate, SA187 was re-isolated from Arabidopsis root system at the end of an initial experiment to confirm the genotype of the inoculated strain. To address the ethylene involvement in Arabidopsis adaptation to salt stress, ACC (1-aminocyclopropane-1-carboxylic acid, Sigma), KMBA (2-keto-4-methylthiobutyric acid, Sigma), AVG (aminoethoxyvinylglycine, Sigma), AgNO_3_ (silver nitrate, Sigma) were added into pre-cooled ½ MS agar medium together with 100 mM NaCl. For DNPH (2,4-dinitrophenylhydrazine, Sigma), 5 mM solution was prepared by solubilizing DNPH into 2M HCl (hydrochloric acid, Sigma) as described previously [81], then the solution was diluted until reaching 1 mM, and equilibrated to the same pH as MS medium (pH 5.8) using 2M KOH (potassium hydroxide, Sigma). DNPH was used at final concentration 3 μM.

All plants were grown in long day conditions in growth chambers (Percival; 16 h light / 8 h dark, 22°C). Each experiment was performed at least in three biological replicates.

### Na^+^ and K^+^ content determination

Dry rosettes and root systems were weighted. All samples were measured individually except for salt-treated root systems, whereby pools of three root systems were measured to ensure proper weight measurements. Sodium and potassium concentrations were prepared for shoot and root dry samples by adding 1 mL of freshly prepared 1% HNO_3_ (nitric acid, Fisher Scientific) to the pre-weighed samples. The concentrations of sodium and potassium were determined, using Inductively Coupled Plasma Optical Emission Spectrometer (Varian 720-ES ICP OES, Australia).

### Generation of GFP-labelled bacteria

SA187 was genetically labeled with the *GFP* expressing cassette by taking advantage of the mini-Tn7 transposon system [82]. In order to specifically select for a bacterium carrying the *GFP* integration in the genome, a spontaneous rifampicin resistant mutant of the strain was obtained first [83]: an overnight-grown culture of SA187 was plated on LB plates supplemented with 100 μg·mL^-1^ of rifampicin, and the plates were incubated for 24 h at 28°C. At least 10 colonies, representing spontaneous rifampicin resistant (Rif^R^) mutants of the strain were streaked twice on LB plates containing 100 μg·mL^-1^ of rifampicin and thereafter twice on LB plates supplemented with 200 μg·mL^-1^ of rifampicin. The GFP expressing cassette was introduced in the SA187 Rif^R^ strain by conjugation as described in Lambertsen et al. (2004) [84]. Briefly, 10^10^ cells of SA187 Rif^R^ strain were mixed with 10^9^ cells of *E*. *coli* SM10λpir harboring the helper plasmid pUX-BF13, the GFP donor (a mini-Tn7) plasmid and mobilizer pRK600 plasmid. The mixed culture was incubated on sterile nitrocellulose filter for 16hrs. The conjugation culture of bacterial cells was resuspended in saline buffer (9 g/L NaCl) and spread on selective media with a propitiate antibiotics to select transformed SA187. The selected colonies were screened by fluorescence microscopy for GFP fluorescence and positive colonies were further subjected to genotype confirmation by 16S rRNA gene sequencing.

### Confocal microscopy

GFP-labeled SA187 on Arabidopsis roots was imaged using an inverted Zeiss LSM 710 confocal microscope equipped with Plan-Apochromat 10x/0.45, Plan-Apochromat 20x/0.8, and Plan-Apochromat 40x/1.4 Oil objectives. Seedlings grown for 3–21 days on vertical ½ MS agar plates or in soil inoculated with SA187-GFP were washed gently in sterile distilled water and transferred on a sterile agar plate. A block of agar with several seedlings was immediately cut out and placed upside-down to a chambered cover glass (Lab-Tek II) with 30 μM propidium iodide (PI) in water as mounting medium. The GFP and PI fluorescence was excited using the 488nm laser line, and captured as a single track (emission of 493–537 nm for the GFP channel, 579–628 nm for the PI channel, 645–708 nm for chloroplast autofluorescence). For 3D reconstructions, 1 μm-step Z-stacks were taken, and images were generated in the integral 3D view of the Zen software (Zeiss).

### Quantification of root colonization

Col-0 seedlings were germinated on SA187-inoculated ½ MS agar plates and transferred to new ½ MS plates with or without 100 mM NaCl 5 days after germination (10 seedlings per plate). Parts of their root systems grown after the transfer were cut, gently washed by dipping in distilled water to remove non-attached bacterial cells, and then ground in Eppendorf tubes using Teflon sticks. Each sample was resuspended in 1 ml of extraction buffer (10 mM MgCl_2_, 0.01% Silwet L-77), sonicated for 1 min and subsequently vortexed for 10 min. Samples were diluted 10-fold, and then spread on LB agar plates, and colony forming units (CFUs) were counted after overnight incubation at 28°C. Calculated number of CFUs was normalized per centimeter of root length (total root length was determined based on images of root systems before their harvest). The experiment was conducted in three biological replicates, each with three technical replicates per condition; each sample consisted of five roots.

### RNA-Seq and qPCR analysis

Total RNA was extracted from 5-day-old plants either or not inoculated with SA187 and transferred for 10 more days on ½ MS plates with or without 100 mM NaCl using the Nucleospin RNA plant kit (Macherey-Nagel), including DNaseI treatment, and following manufacturer’s recommendations.

RNA samples were analyzed by Illumina HiSeq deep sequencing (Illumina HiSeq 2000, Illumina). Three biological replicates were processed for each sample. Paired-end sequencing of RNA-Seq samples was performed using Illumina GAIIx with a read length of 100 bp. Reads were quality-controlled using FASTQC (http://www.bioinformatics.babraham.ac.uk/projects/fastqc/). Trimmomatic was used for quality trimming [85]. Parameters for read quality filtering were set as follows: Minimum length of 36 bp; Mean Phred quality score greater than 30; Leading and trailing bases removal with base quality below 3; Sliding window of 4:15. TopHat v2.0.9 [86] was used for alignment of short reads to the *A*. *thaliana* genome TAIR10, Cufflinks v2.2.0 [87] for transcript assembly and differential expression. To identify differentially expressed genes, specific parameters (*p*-value: 0.05; statistical correction: Benjamini Hochberg; FDR: 0.05) in cuffdiff were used. Post-processing and visualization of differential expression were done using cummeRbund v2.0.0 [88]. Gene was considered as regulated if fold change > log_2_^|0.6|^ and *q*-value < 0.05 compared to Mock condition. RNA-Seq data set can be retrieved under NCBI geo submission ID GSE102950.

For qPCR analysis, mock and SA187-inoculated plants were used for RNA extraction as described above. Samples were used for analysis of either plant or SA187 gene expression. For bacteria alone, SA187 incubated for 4h in liquid ½ MS or ½ MS with 100 mM NaCl at 28°C and dark were used for RNA extraction, using the RiboPure RNA Purification Kit (Ambion), following manual instructions for Gram-negative bacteria, with the exception that no beads were added during bacterial lysis. RNA extraction was followed by DNAseI treatment.

cDNAs were using SuperscriptIII (Invitrogen): 1 μg of total RNA, oligo-dT as a primer, following manufacturer’s recommendations. For Arabidopsis gene expression analyses, *ACTIN2* (At3g18780) and *UBIQUITIN10* (At4g05320) were used as reference genes. For SA187 gene expression analyses, *infB*, *rpoB* and *gyrB* were used as reference genes. All reactions were done in a CFX96 Touch Real-Time PCR Detection System (BIO-RAD) as follows: 50°C for 2 min, 95°C for 10 min; 40× [95°C for 10 sec and 60°C for 40 sec]; and a dissociation step to validate PCR products. All reactions were performed in three biological replicates, and each reaction as a technical triplicate. Gene expression levels were calculated using the Bio-Rad CFX manager software. Primer sequences used in this analysis are listed in S3 Table.

### Hierarchical clustering and gene family enrichment

Arabidopsis regulated genes were used to generate HCL tree using Multi Experiment Viewer (MeV 4.9.0 version, TM4, https://sourceforge.net/projects/mev-tm4/files/mev-tm4/MeV%204.9.0/). Raw data were normalized for every gene. Hierarchical clustering was performed using Euclidian distances, average linkage and leaf order optimization.

Gene enrichment analyses were performed using AmiGO website (http://amigo1.geneontology.org/cgi-bin/amigo/term_enrichment). All clusters were analyzed using default parameter (S2 Table).

### Hormone content analysis

For each sample, 10 mg of freeze-dried powder were extracted with 0.8 mL of acetone/water/acetic acid (80/19/1 v:v:v). For each sample, 2 ng of each standard was added to the sample: abscisic acid, salicylic acid, jasmonic acid, and indole-3-acetic acid stable labeled isotopes used as internal standards were prepared as described previously [89]. The extract was vigorously shaken for 1 min, sonicated for 1 min at 25 Hz, shaken for 10 minutes at 4°C in a Thermomixer (Eppendorf), and then centrifuged (8000 g, 4°C, 10 min). The supernatants were collected, and the pellets were re-extracted twice with 0.4 mL of the same extraction solution, then vigorously shaken (1 min) and sonicated (1 min; 25 Hz). After the centrifugations, three supernatants were pooled and dried.

Each dry extract was dissolved in 140 μL of acetonitrile/water (50/50; v/v), filtered, and analyzed using a Waters Acquity ultra performance liquid chromatograph coupled to a Waters Xevo Triple quadrupole mass spectrometer TQS (UPLC-ESI-MS/MS). The compounds were separated on a reverse-phase column (Uptisphere C18 UP3HDO, 100 × 2.1 mm, 3 μm particle size; Interchim, France) using a flow rate of 0.4 mL·min^-1^ and a binary gradient: (A) acetic acid 0.1% in water (v/v) and (B) acetonitrile with 0.1% acetic acid. For ABA, salicylic acid, jasmonic acid, the following binary gradients were used (time, % A): (0 min, 98%), (3 min, 70%), (7.5 min, 50%), (8.5 min, 5%), (9.6 min, 0%), (13.2 min, 98%), (15.7 min, 98%), and the column temperature was 40°C. Mass spectrometry was conducted in electrospray and multiple reaction monitoring scanning mode (MRM mode), in the negative ion mode. Relevant instrumental parameters were set as follows: capillary 1.5 kV (negative mode), source block and desolvation gas temperatures 130°C and 500°C, respectively. Nitrogen was used to assist the cone and desolvation (150 L·h^-1^ and 800 L·h^-1^, respectively), argon was used as the collision gas at a flow of 0.18 mL·min^-1^. Samples were reconstituted in 140 μL of 50/50 acetonitrile/H_2_O (v/v) per mL of injected volume. The limit of detection (LOD) and limit of quantification (LOQ) were extrapolated for each hormone from calibration curves and samples using Quantify module of MassLynx software, version 4.1.

### GUS staining

Seedlings were vacuum infiltrated with the pre-fixation buffer [0.3% formaldehyde, 0.28% mannitol, 50 mM sodium phosphate buffer (pH 7.2)], washed with phosphate buffer and incubated in staining solution [250 μM K_3_Fe(CN)_6_ (potassium ferricyanide), 250 μM K_4_Fe(CN)_6_ (potassium ferrocyanide), 2% Triton-X, 1 mM 5-bromo-4-chloro-3-indolyl-b-D-glucuronic acid (X-GlcA; Duchefa), 50 mM sodium phosphate buffer (pH 7.2)]. Tissue was cleared with Visokol (Phytosys) overnight and observed with Axio Imager 2 (Zeiss) equipped with Plan-Neofluar 10x/0.45 objective.

### Measurement of *in vitro* ethylene emanation

A fresh SA187 culture was prepared by inoculation of 50 mL of liquid LB medium with 1 mL of overnight-grown culture. Subsequently, 2 mL of fresh culture was transferred to 10 mL chromatography vials and sealed with a rubber plug and snap-cap (Chromacol) after 0, 1, 2 or 4 hours of growth on a shaker incubator (220 rpm, 28°C). The sealed vials were again transferred to the shaker incubator for another 2 hours to allow ethylene accumulation. Three biological replicates were prepared at each time point along with 3 controls to correct for background ethylene emanation. Ethylene emission was measured with a laser-based photo-acoustic detector (ETD-300 ethylene detector, Sensor Sense, The Netherlands) [90]. Immediately after the ethylene measurement, OD_600_ was determined with Implen NanoPhotometer NP80 (Sopachem Life Sciences, Belgium) to correct for the total amount of bacterial cells present in the samples.

### Data submission

RNA-Seq data are available under the ID GSE102950 (http://www.ncbi.nlm.nih.gov/geo/query/acc.cgi?acc=GSE102950)

## Supporting information

S1 Fig Meteorological data for field trials in Hada Al-Sham. Precipitations and maximal/minimal temperature recorded in experimental agriculture facility in Hada Al-Sham where field trials with alfalfa were conducted in seasons 2015–16 and 2016–17.
(PDF)

S2 Fig Scheme of SA187 inoculation and plant treatments. Sterilized seeds were placed on agar plates containing either ½ MS or ½ MS + SA187 (2·10^5^ cells/ml), defining the mock- and SA187-inoculated plants, respectively. Five days after germination, mock and SA187-inoculated were transferred on control agar plates (½ MS) or stress agar plates (containing 100 mM NaCl or PEG) to evaluate plant tolerance to abiotic stresses.
(PDF)

S3 Fig Root hair length of 16-day-old seedlings. Average root hair length of 10% longest root hairs (n > 100) in 16-day-old seedlings grown vertically on ½ MS medium with or 100 mM NaCl. Seedlings were transferred 5 days after germination from ½ MS agar plates without (mock) or with SA187. Only root hairs emerged after the seedling transfer were measured. Error bars represent SD. Asterisks indicate a statistical difference based on the Student t-test (*** P < 0.001).
(PDF)

S4 Fig The effect of inactivated and GFP-tagged SA187 on Arabidopsis growth. (A) Fresh weight of 17-day-old Arabidopsis seedlings exposed to salt stress (½ MS + 100mM NaCl) for 12 days in the presence of heat-inactivated SA187 in comparison to living SA187. (B) Fresh weight of 17-day-old Arabidopsis seedlings exposed to salt stress (½ MS + 100mM NaCl) for 12 days colonized by GFP-tagged SA187 in comparison to wild-type SA187. Error bars represent SE. Asterisks indicate a statistical difference to Mock based on the Student’s t-test (* P < 0.05; ** P < 0.01, *** P < 0.001). No significant difference was recorded between SA187 and SA187-GFP (at P < 0.05).
(PDF)

S5 Fig Quantification of root colonization by SA187. Efficiency of root colonization evaluated by counting colony forming units (CFU) and normalized per root centimeter. Seedlings were grown on ½ MS medium (Control) or ½ MS with 100 mM NaCl for 5 days. Bars represent SE, n = 9, each sample consists of 5 roots. Asterisks indicate a statistical difference based on the Student’s t-test (*** P < 0.001).
(PDF)

S6 Fig Growth of SA187-treated Arabidopsis mutants in hormonal pathways under normal conditions. Fresh weight (mg) of SA187-colonized plants after growth on ½ MS for 17 days. All plots represent the mean of three biological replicates (n > 36). Error bars represent SE. ^*+*^ *acs* represents the heptuple mutant *acs1-1 acs2-1 acs4-1 acs5-2 acs6-1 acs7-1 acs9-1*, and *pyr1/pyl* the quadruple mutant *pyr1 pyl1 pyl2 pyl4*.
(PDF)

S7 Fig The effect of ACC and KMBA treatment on Arabidopsis growth in comparison to SA187-inoculated seedlings. Complete data to spider graphs in Figs 7C and 8B. Fresh weight (A), root length (B), and lateral root density (C) of 17-day-old seedlings grown on ½ MS + 100 mM NaCl for the last 12 days. Values represent means of three biological experiments, each in two technical replicates (> 33 seedlings). Error bars represent SE. Asterisks indicate a statistical difference from mock-inoculated plants based on Student’s t-test (* P < 0.05; ** P < 0.01; *** P <0.001).
(PDF)

S8 Fig Ethylene emission by SA187 on synthetic medium. (A) Ethylene emission of in vitro SA187 cultures at different stages after inoculation. Average OD600 values at each time point are given. Grey bar: LB medium without SA187; Green bars: LB medium with SA187. (B) Ethylene emission corrected for background ethylene levels emitted by controls and standardized per unit OD600. Measurements based on 3 biological replicates per time point. Experiment was repeated three times with similar results; a representative experiment is shown. Error bars represent SD. For (A) no significant differences were found between each time point versus the control based on the Mann-Whitney U test (P < 0.05).
(PDF)

S1 Table RNAseq analysis. Whole genome analysis based on TAIR10 annotation. For each comparison, log_2_ fold change is indicated as well as adjusted p-value (q-value).
(XLSX)

S2 Table GO term analysis. GO term significant enrichment for every cluster. Gene enrichment analyses were performed using AmiGO1 website (http://amigo1.geneontology.org/cgi-bin/amigo/term_enrichment), using default parameter.
(XLSX)

S3 Table Primers used during this study. (PPTX)
