# Supplementary material for: Ethylene induced plant stress tolerance by Enterobacter sp. SA187 is mediated by 2‐keto‐4‐methylthiobutyric acid production
Source: PLoS Genet. 2018 Mar 19;14(3):e1007273. doi: 10.1371/journal.pgen.1007273 (PMC5875868; doi:10.1371/journal.pgen.1007273)
Supplement: S1 Fig — Precipitations and maximal/minimal temperature recorded in experimental agriculture facility in Hada Al-Sham where field trials with alfalfa were conducted in seasons 2015–16 and 2016–17. (PDF) [file pgen.1007273.s001.pdf]

Supplemental Figures

Plant salt stress tolerance by the desert endophyte *Enterobacter* sp. SA187 is mediated through bacterially produced 2-keto-4-methylthiobutyric acid stimulating the ethylene signaling

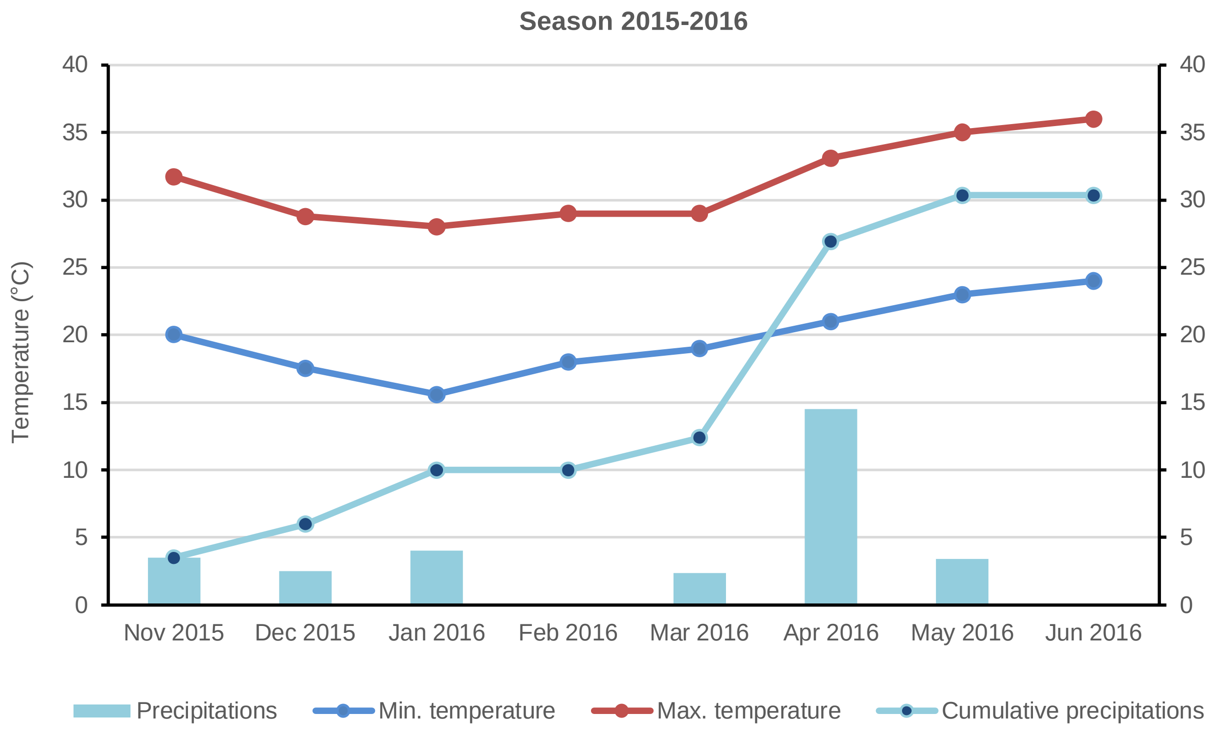

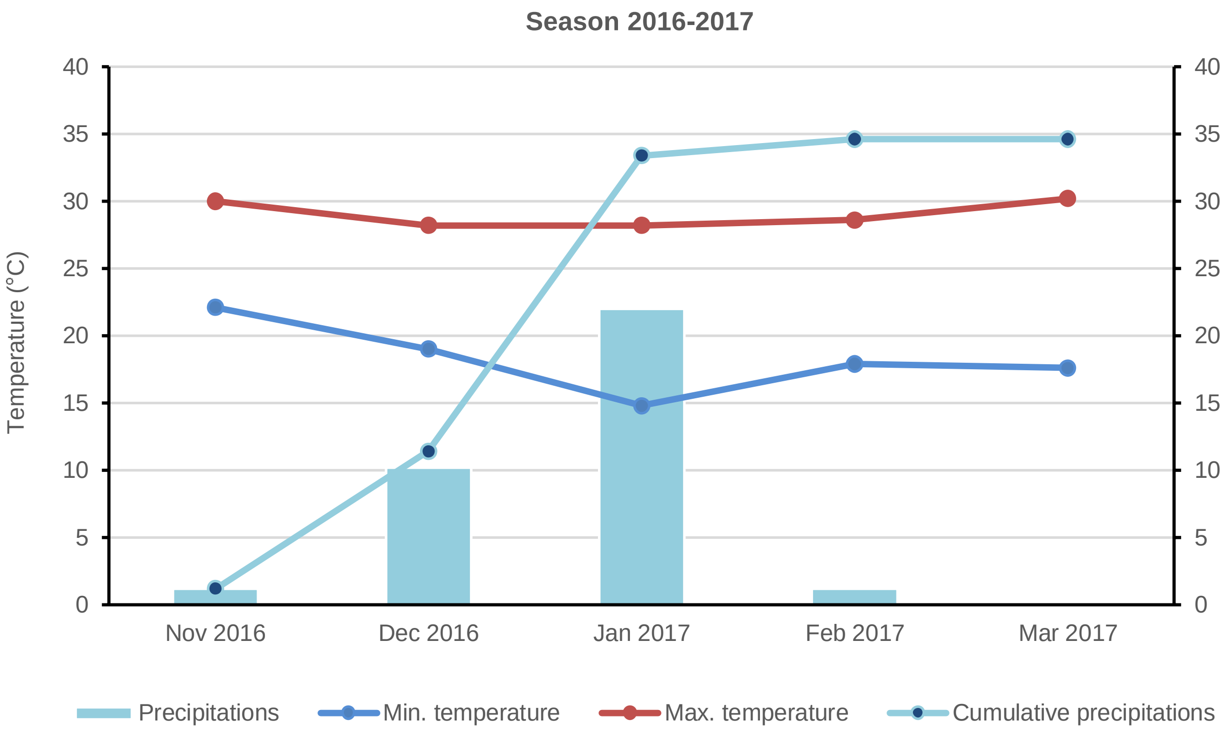

Figure S1. Meteorological data for fields trials in Hada Al-Sham.

Precipitations, and maximal/minimal temperature recorded in the experimental agriculture facility in Hada Al-Sham (Saudi Arabia) where field trials with alfalfa were conducted in seasons 2015-16 and 2016-17.
