# Supplementary material for: Ethylene induced plant stress tolerance by Enterobacter sp. SA187 is mediated by 2‐keto‐4‐methylthiobutyric acid production
Source: PLoS Genet. 2018 Mar 19;14(3):e1007273. doi: 10.1371/journal.pgen.1007273 (PMC5875868; doi:10.1371/journal.pgen.1007273)
Supplement: S2 Fig — Sterilized seeds were placed on agar plates containing either ½ MS or ½ MS + SA187 (2·105 cells/ml), defining the mock- and SA187-inoculated plants, respectively. Five days after germination, mock and SA187-inoculated were transferred on control agar plates (½ MS) or stress agar plates (containing 100 mM NaCl or PEG) to evaluate plant tolerance to abiotic stresses. (PDF) [file pgen.1007273.s002.pdf]

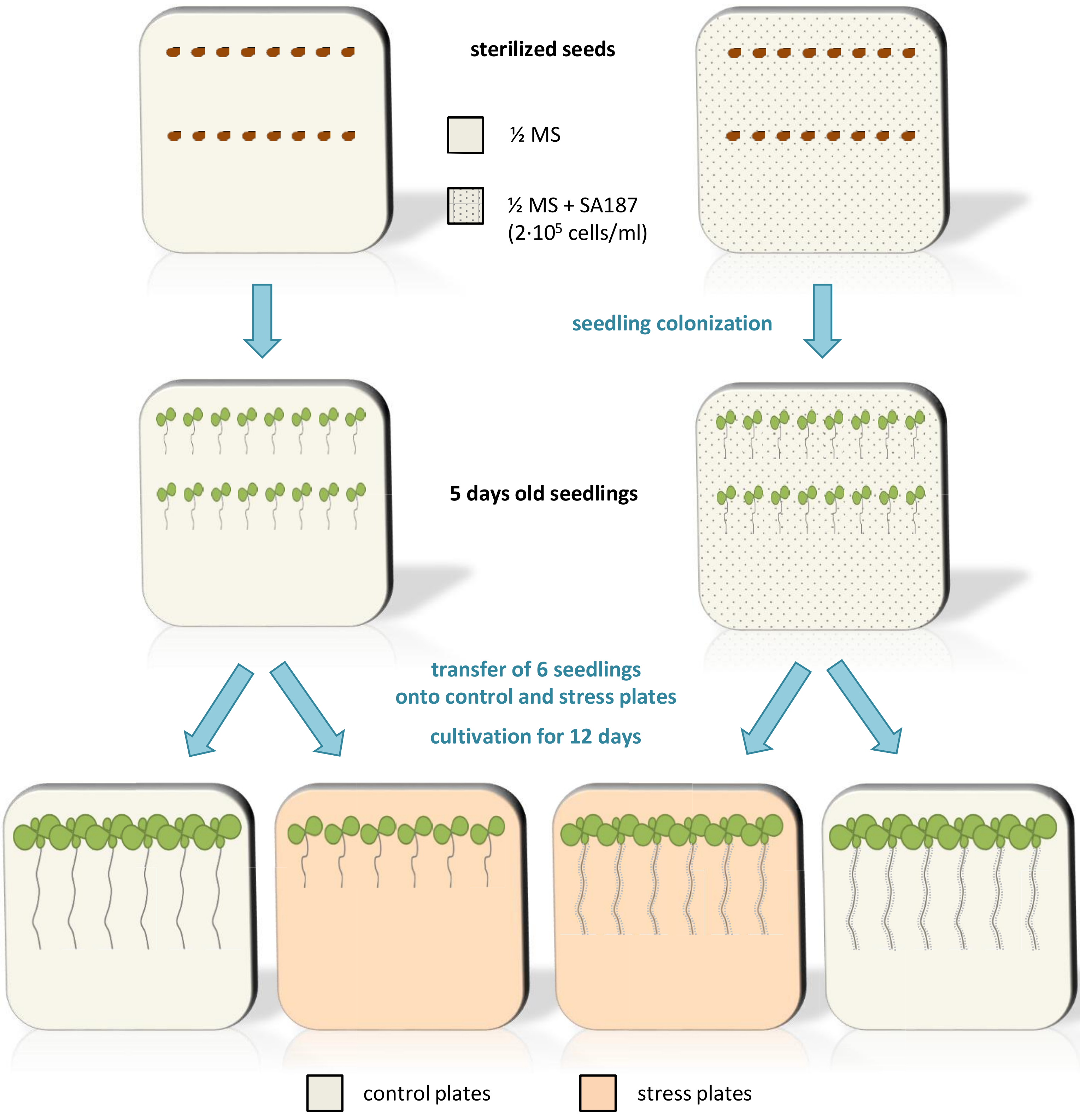

Figure S2. Scheme of the SA187 inoculation and plant treatments.

Sterilized seeds were placed on agar plates containing either ½ MS or ½ MS + SA187 ($2 \cdot 10^5$ cells/ml), defining the mock- and SA187-inoculated plants, respectively. Five days after germination, mock- and SA187-inoculated seedlings were transferred onto control agar plates (½ MS) or salt-stress agar plates (containing 100 mM NaCl) to evaluate plant tolerance to the salt stress.
