# Supplementary material for: Ethylene induced plant stress tolerance by Enterobacter sp. SA187 is mediated by 2‐keto‐4‐methylthiobutyric acid production
Source: PLoS Genet. 2018 Mar 19;14(3):e1007273. doi: 10.1371/journal.pgen.1007273 (PMC5875868; doi:10.1371/journal.pgen.1007273)
Supplement: S3 Fig — Average root hair length of 10% longest root hairs (n > 100) in 16-day-old seedlings grown vertically on ½ MS medium with or 100 mM NaCl. Seedlings were transferred 5 days after germination from ½ MS agar plates without (mock) or with SA187. Only root hairs emerged after the seedling transfer were measured. Error bars represent SD. Asterisks indicate a statistical difference based on the Student t-test (*** P < 0.001). (PDF) [file pgen.1007273.s003.pdf]

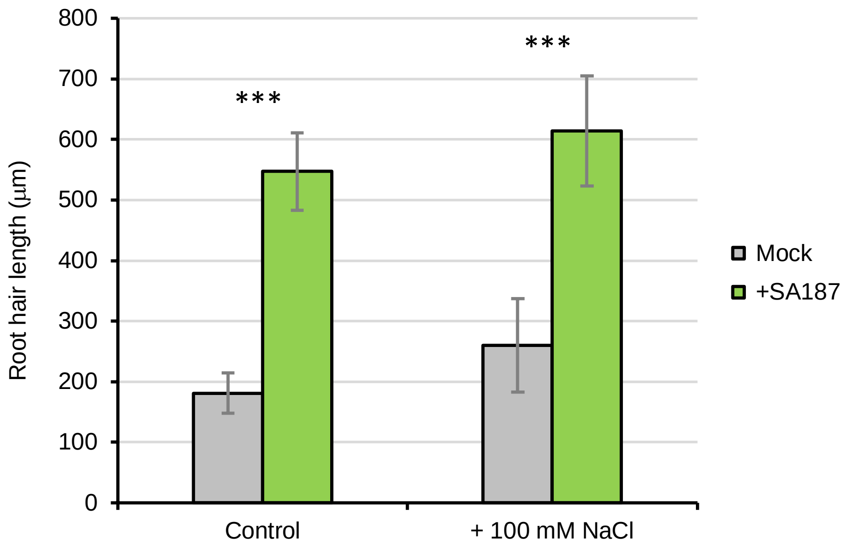

Figure S3. Root hair length of 16-day-old seedlings.

Average root hair length of 10% longest root hairs (n > 100) of 16-day-old seedlings grown vertically on ½ MS medium with or without 100 mM NaCl. Seedlings were transferred to such agar plates 5 days after germination from ½ MS agar plates without (Mock) or with SA187. Only root hairs that emerged after the seedling transfer were measured. Error bars represent SD. Asterisks indicate a statistical difference based on the Student's t-test (*** $P < 0.001$).
