# Supplementary material for: Ethylene induced plant stress tolerance by Enterobacter sp. SA187 is mediated by 2‐keto‐4‐methylthiobutyric acid production
Source: PLoS Genet. 2018 Mar 19;14(3):e1007273. doi: 10.1371/journal.pgen.1007273 (PMC5875868; doi:10.1371/journal.pgen.1007273)
Supplement: S4 Fig — (A) Fresh weight of 17-day-old Arabidopsis seedlings exposed to salt stress (½ MS + 100mM NaCl) for 12 days in the presence of heat-inactivated SA187 in comparison to living SA187. (B) Fresh weight of 17-day-old Arabidopsis seedlings exposed to salt stress (½ MS + 100mM NaCl) for 12 days colonized by GFP-tagged SA187 in comparison to wild-type SA187. Error bars represent SE. Asterisks indicate a statistical difference to Mock based on the Student’s t-test (* P < 0.05; ** P < 0.01, *** P < 0.001). No significant difference was recorded between SA187 and SA187-GFP (at P < 0.05). (PDF) [file pgen.1007273.s004.pdf]

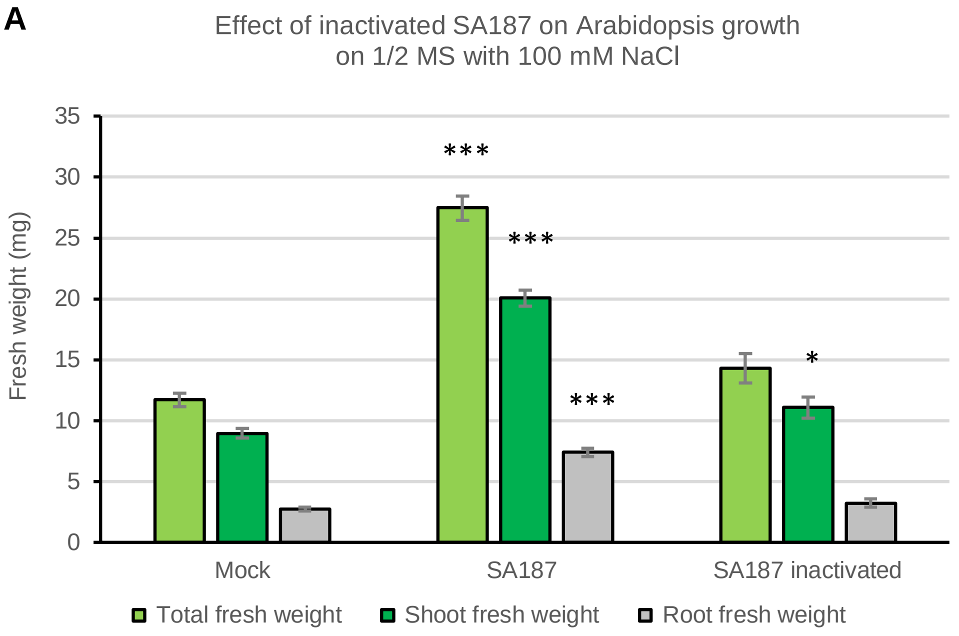

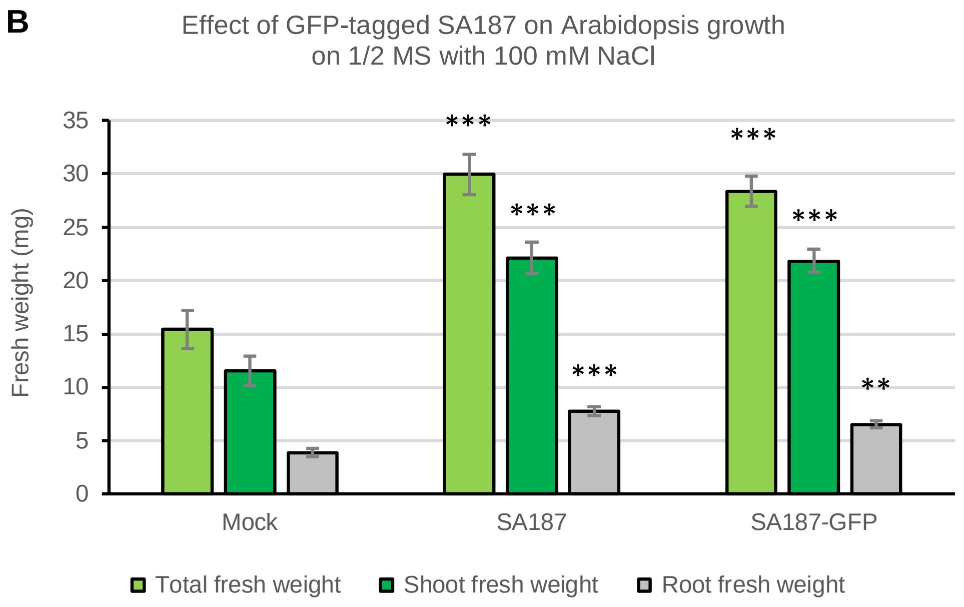

Figure S4. The effect of inactivated and GFP-tagged SA187 on Arabidopsis growth.

(A) Fresh weight of 17-day-old Arabidopsis seedlings exposed to salt stress (½ MS + 100mM NaCl) for 12 days in the presence of heat-inactivated SA187 in comparison to living SA187. Error bars represent SE. Asterisks indicate a statistical difference to Mock based on the Student's t-test (* $P < 0.05$; ** $P < 0.01$; *** $P < 0.001$).

(B) Fresh weight of 17-day-old Arabidopsis seedlings exposed to salt stress (½ MS + 100mM NaCl) for 12 days colonized by GFP-tagged SA187 in comparison to wild-type SA187. No significant difference was recorded between SA187 and SA187-GFP. Error bars represent SE. Asterisks indicate a statistical difference to Mock based on the Student's t-test (* $P < 0.05$; ** $P < 0.01$; *** $P < 0.001$).
