# Supplementary material for: Ethylene induced plant stress tolerance by Enterobacter sp. SA187 is mediated by 2‐keto‐4‐methylthiobutyric acid production
Source: PLoS Genet. 2018 Mar 19;14(3):e1007273. doi: 10.1371/journal.pgen.1007273 (PMC5875868; doi:10.1371/journal.pgen.1007273)
Supplement: S5 Fig — Efficiency of root colonization evaluated by counting colony forming units (CFU) and normalized per root centimeter. Seedlings were grown on ½ MS medium (Control) or ½ MS with 100 mM NaCl for 5 days. Bars represent SE, n = 9, each sample consists of 5 roots. Asterisks indicate a statistical difference based on the Student’s t-test (*** P < 0.001). (PDF) [file pgen.1007273.s005.pdf]

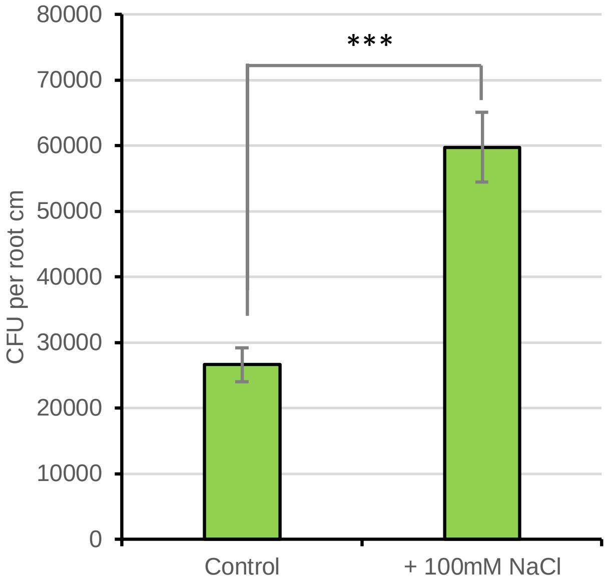

Figure S5. Quantification of root colonization by SA187.

Efficiency of root colonization evaluated by counting colony forming units (CFU) and normalized per root centimeter. Seedlings were grown on ½ MS medium (Control) or ½ MS with 100 mM NaCl for 5 days. Bars represent SE, n = 9, each sample consists of 5 roots. Asterisks indicate a statistical difference based on the Student’s t-test (*** $P < 0.001$).
