# Supplementary material for: Ethylene induced plant stress tolerance by Enterobacter sp. SA187 is mediated by 2‐keto‐4‐methylthiobutyric acid production
Source: PLoS Genet. 2018 Mar 19;14(3):e1007273. doi: 10.1371/journal.pgen.1007273 (PMC5875868; doi:10.1371/journal.pgen.1007273)
Supplement: S6 Fig — Fresh weight (mg) of SA187-colonized plants after growth on ½ MS for 17 days. All plots represent the mean of three biological replicates (n > 36). Error bars represent SE. + acs represents the heptuple mutant acs1-1 acs2-1 acs4-1 acs5-2 acs6-1 acs7-1 acs9-1, and pyr1/pyl the quadruple mutant pyr1 pyl1 pyl2 pyl4. (PDF) [file pgen.1007273.s006.pdf]

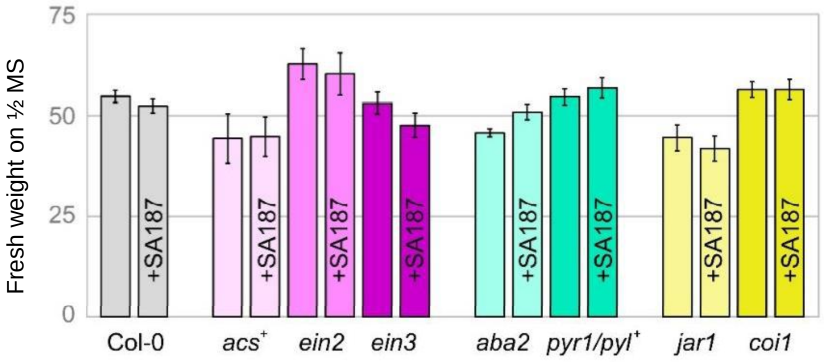

Figure S6. Growth of SA187-treated Arabidopsis mutants in hormonal pathways under normal conditions.

Fresh weight (mg) of mock- and SA187-inoculated plants after growth on ½ MS for 17 days.

$^{+}$ *acs* represents the heptuple mutant *acs1-1 acs2-1 acs4-1 acs5-2 acs6-1 acs7-1 acs9-1*, and *pyr1/pyl* the quadruple mutant *pyr1 pyl1 pyl2 pyl4*.
