# Supplementary material for: Ethylene induced plant stress tolerance by Enterobacter sp. SA187 is mediated by 2‐keto‐4‐methylthiobutyric acid production
Source: PLoS Genet. 2018 Mar 19;14(3):e1007273. doi: 10.1371/journal.pgen.1007273 (PMC5875868; doi:10.1371/journal.pgen.1007273)
Supplement: S7 Fig — Complete data to spider graphs in Figs 7C and 8B. Fresh weight (A), root length (B), and lateral root density (C) of 17-day-old seedlings grown on ½ MS + 100 mM NaCl for the last 12 days. Values represent means of three biological experiments, each in two technical replicates (> 33 seedlings). Error bars represent SE. Asterisks indicate a statistical difference from mock-inoculated plants based on Student’s t-test (* P < 0.05; ** P < 0.01; *** P <0.001). (PDF) [file pgen.1007273.s007.pdf]

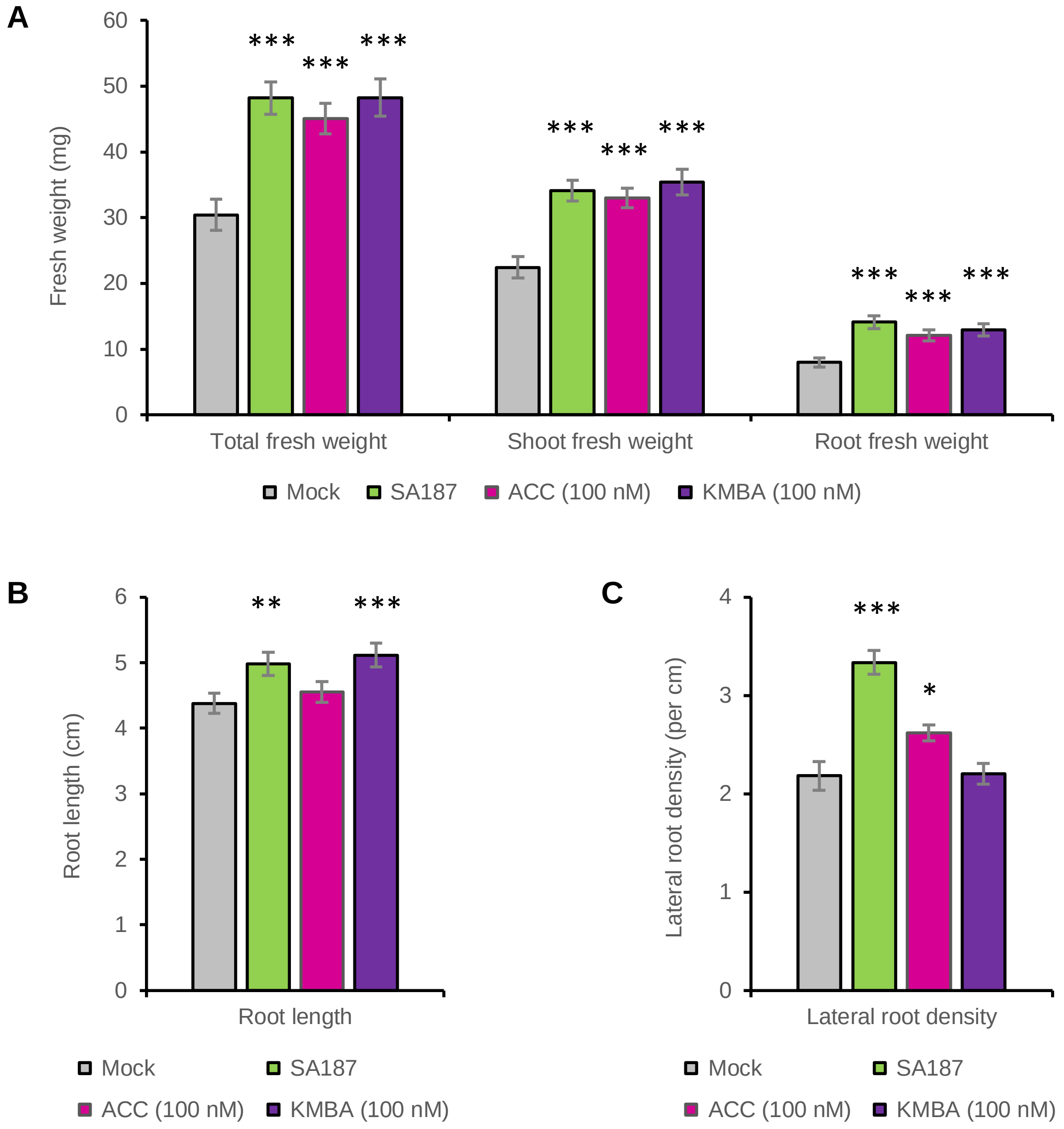

Figure S7. The effect of ACC and KMBA treatment on Arabidopsis growth in comparison to SA187-inoculated seedlings.

Complete data to spider graphs in Figure 7C and Figure 8B. Fresh weight (A), root length (B), and lateral root density (C) of 17-day-old seedlings grown on ½ MS + 100 mM NaCl for the last 12 days. Values represent means of three biological experiments, each in two technical replicates (> 33 seedlings). Error bars represent SE. Asterisks indicate a statistical difference from mock-inoculated plants based on Student t-test (* $P < 0.05$; ** $P < 0.01$; *** $P < 0.001$).
