# Supplementary material for: Ethylene induced plant stress tolerance by Enterobacter sp. SA187 is mediated by 2‐keto‐4‐methylthiobutyric acid production
Source: PLoS Genet. 2018 Mar 19;14(3):e1007273. doi: 10.1371/journal.pgen.1007273 (PMC5875868; doi:10.1371/journal.pgen.1007273)
Supplement: S8 Fig — (A) Ethylene emission of in vitro SA187 cultures at different stages after inoculation. Average OD600 values at each time point are given. Grey bar: LB medium without SA187; Green bars: LB medium with SA187. (B) Ethylene emission corrected for background ethylene levels emitted by controls and standardized per unit OD600. Measurements based on 3 biological replicates per time point. Experiment was repeated three times with similar results; a representative experiment is shown. Error bars represent SD. For (A) no significant differences were found between each time point versus the control based on the Mann-Whitney U test (P < 0.05). (PDF) [file pgen.1007273.s008.pdf]

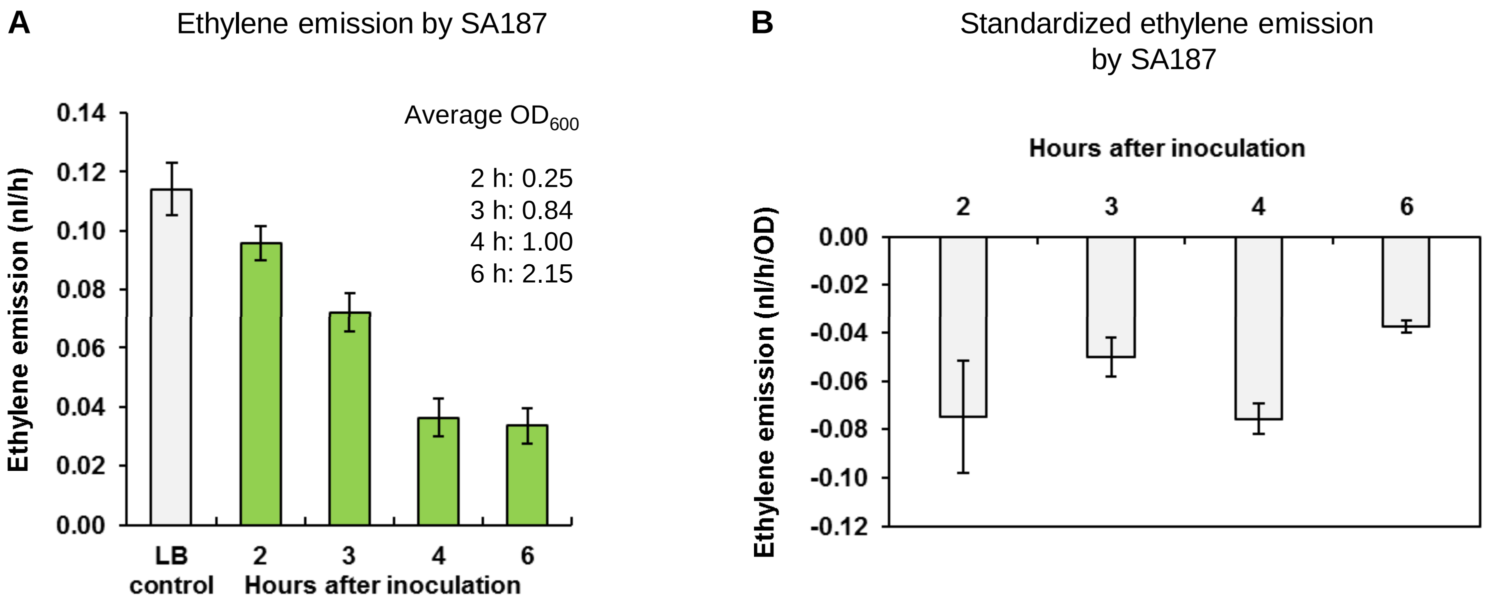

Figure S8. Ethylene emission by SA187 on synthetic medium.

(A) Ethylene emission of *in vitro* SA187 cultures at different stages after inoculation. Average OD_{600} values at each time point are given. Grey bar: LB medium without SA187; Green bars: LB medium with SA187. (B) Ethylene emission corrected for background ethylene levels emitted by controls and standardized per unit OD_{600}.
Measurements based on 3 biological replicates per time point. Experiment was repeated three times with similar results; a representative experiment is shown. Error bars represent SD. For (A) no significant differences were found between each time point versus the control based on the Mann-Whitney U test ($P < 0.05$).
