# Supplementary material for: Ethylene induced plant stress tolerance by Enterobacter sp. SA187 is mediated by 2‐keto‐4‐methylthiobutyric acid production
Source: PLoS Genet. 2018 Mar 19;14(3):e1007273. doi: 10.1371/journal.pgen.1007273 (PMC5875868; doi:10.1371/journal.pgen.1007273)
Supplement: S3 Table — (PPTX) [file pgen.1007273.s011.pptx]

## Slide 1
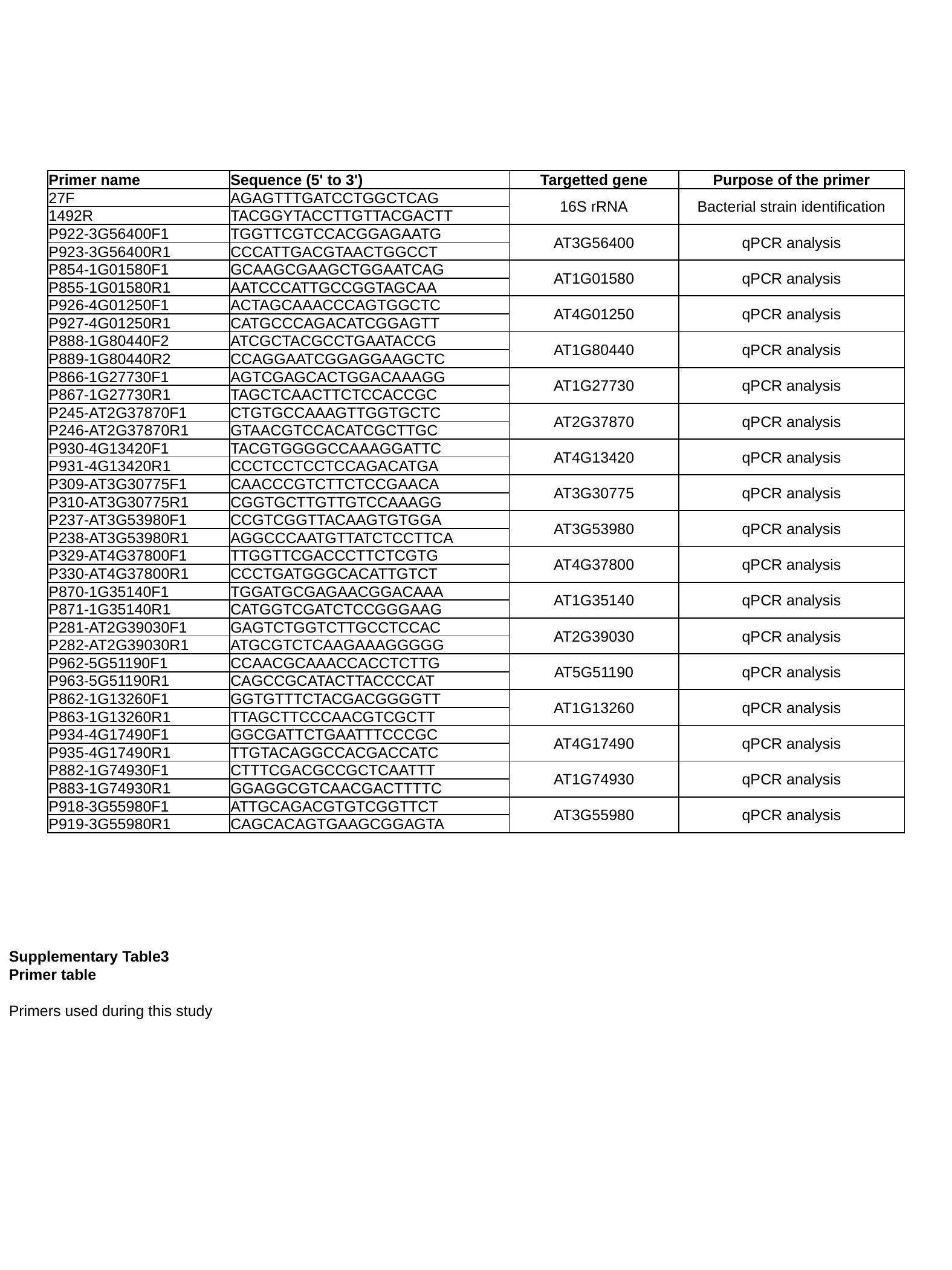

| Primer name | Sequence (5' to 3') | Targetted gene | Purpose of the primer |
| --- | --- | --- | --- |
| 27F | AGAGTTTGATCCTGGCTCAG | 16S rRNA | Bacterial strain identification |
| 1492R | TACGGYTACCTTGTTACGACTT | | |
| P922-3G56400F1 | TGGTTCGTCCACGGAGAATG | AT3G56400 | qPCR analysis |
| P923-3G56400R1 | CCCATTGACGTAACTGGCCT | | |
| P854-1G01580F1 | GCAAGCGAAGCTGGAATCAG | AT1G01580 | qPCR analysis |
| P855-1G01580R1 | AATCCCATTGCCGGTAGCAA | | |
| P926-4G01250F1 | ACTAGCAAACCCAGTGGCTC | AT4G01250 | qPCR analysis |
| P927-4G01250R1 | CATGCCCAGACATCGGAGTT | | |
| P888-1G80440F2 | ATCGCTACGCCTGAATACCG | AT1G80440 | qPCR analysis |
| P889-1G80440R2 | CCAGGAATCGGAGGAAGCTC | | |
| P866-1G27730F1 | AGTCGAGCACTGGACAAAGG | AT1G27730 | qPCR analysis |
| P867-1G27730R1 | TAGCTCAACTTCTCCACCGC | | |
| P245-AT2G37870F1 | CTGTGCCAAAGTTGGTGCTC | AT2G37870 | qPCR analysis |
| P246-AT2G37870R1 | GTAACGTCCACATCGCTTGC | | |
| P930-4G13420F1 | TACGTGGGGCCAAAGGATTC | AT4G13420 | qPCR analysis |
| P931-4G13420R1 | CCCTCCTCCTCCAGACATGA | | |
| P309-AT3G30775F1 | CAACCCGTCTTCTCCGAACA | AT3G30775 | qPCR analysis |
| P310-AT3G30775R1 | CGGTGCTTGTTGTCCAAAGG | | |
| P237-AT3G53980F1 | CCGTCGGTTACAAGTGTGGA | AT3G53980 | qPCR analysis |
| P238-AT3G53980R1 | AGGCCCAATGTTATCTCCTTCA | | |
| P329-AT4G37800F1 | TTGGTTCGACCCTTCTCGTG | AT4G37800 | qPCR analysis |
| P330-AT4G37800R1 | CCCTGATGGGCACATTGTCT | | |
| P870-1G35140F1 | TGGATGCGAGAACGGACAAA | AT1G35140 | qPCR analysis |
| P871-1G35140R1 | CATGGTCGATCTCCGGGAAG | | |
| P281-AT2G39030F1 | GAGTCTGGTCTTGCCTCCAC | AT2G39030 | qPCR analysis |
| P282-AT2G39030R1 | ATGCGTCTCAAGAAAGGGGG | | |
| P962-5G51190F1 | CCAACGCAAACCACCTCTTG | AT5G51190 | qPCR analysis |
| P963-5G51190R1 | CAGCCGCATACTTACCCCAT | | |
| P862-1G13260F1 | GGTGTTTCTACGACGGGGTT | AT1G13260 | qPCR analysis |
| P863-1G13260R1 | TTAGCTTCCCAACGTCGCTT | | |
| P934-4G17490F1 | GGCGATTCTGAATTTCCCGC | AT4G17490 | qPCR analysis |
| P935-4G17490R1 | TTGTACAGGCCACGACCATC | | |
| P882-1G74930F1 | CTTTCGACGCCGCTCAATTT | AT1G74930 | qPCR analysis |
| P883-1G74930R1 | GGAGGCGTCAACGACTTTTC | | |
| P918-3G55980F1 | ATTGCAGACGTGTCGGTTCT | AT3G55980 | qPCR analysis |
| P919-3G55980R1 | CAGCACAGTGAAGCGGAGTA | | |
Supplementary Table3
Primer table
Primers used during this study
